# The Biocontrol and Plant Growth-Promoting Properties of *Streptomyces alfalfae* XN-04 Revealed by Functional and Genomic Analysis

**DOI:** 10.3389/fmicb.2021.745766

**Published:** 2021-09-22

**Authors:** Jing Chen, Lifang Hu, Na Chen, Ruimin Jia, Qing Ma, Yang Wang

**Affiliations:** College of Plant Protection, Northwest A&F University, Yangling, China

**Keywords:** *Fusarium oxysporum* f. sp. *vasinfectum*, *Streptomyces alfalfae*, antifungal, plant growth-promoting, genome

## Abstract

Fusarium wilt of cotton, caused by the pathogenic fungal *Fusarium oxysporum* f. sp. *vasinfectum* (*Fov*), is a devastating disease of cotton, dramatically affecting cotton production and quality. With the increase of pathogen resistance, controlling Fusarium wilt disease has become a significant challenge. Biocontrol agents (BCAs) can be used as an additional solution to traditional crop breeding and chemical control. In this study, an actinomycete with high inhibitory activity against *Fov* was isolated from rhizosphere soil and identified as *Streptomyces alfalfae* based on phylogenetic analyses. Next, an integrative approach combining genome mining and metabolites detection was applied to decipher the significant biocontrol and plant growth-promoting properties of XN-04. Bioinformatic analysis and bioassays revealed that the antagonistic activity of XN-04 against *Fov* was associated with the production of various extracellular hydrolytic enzymes and diffusible antifungal metabolites. Genome analysis revealed that XN-04 harbors 34 secondary metabolite biosynthesis gene clusters. The ability of XN-04 to promote plant growth was correlated with an extensive set of genes involved in indoleacetic acid biosynthesis, 1-aminocyclopropane-1-carboxylic acid deaminase activity, phosphate solubilization, and iron metabolism. Colonization experiments indicated that EGFP-labeled XN-04 had accumulated on the maturation zones of cotton roots. These results suggest that *S. alfalfae* XN-04 could be a multifunctional BCA and biofertilizer used in agriculture.

## Introduction

Fusarium wilt of cotton, caused by the pathogen *Fusarium oxysporum* f. sp. *vasinfectum* (*Fov*) W.C. Synder and H.N. Hans, has led to severe losses in yield and quality in most cotton-growing areas of the world (Guo et al., 2015). The hyphae of *Fov* reside in the woody vascular tissues and produce chlamydospores which are able to persist in soil for over 10years (Cox et al., 2019). Therefore, it is quite difficult to control Fusarium wilt of cotton. The current strategies for the management of Fusarium wilt include breeding disease-resistant varieties, seed and soil disinfection, rotation, and chemical control (Cianchetta and Davis, 2015). In fact, Fusarium wilt management relies mainly on synthetic antifungal agents (e.g., carbendazim and azoxystrobin). Although chemical fungicide treatment shows some reduction of Fusarium wilt, the long-term overuse of fungicides has been reported to produce plenty of adverse effects, e.g., serious environmental pollution, threats to animal and human health, poor soil quality, and pathogens resistance to fungicide (Raza et al., 2017). Consequently, it is important to develop alternative methods and agents which have low toxicity and are more environmentally friendly in efficient control of Fusarium wilt. Among the alternatives, biological control may be one of the few options that show potential (Chen et al., 2018).

A number of biocontrol agents (BCAs), such as *Trichoderma* spp. (Nieto-Jacobo et al., 2017; Hewedy et al., 2020; Diaz-Gutierrez et al., 2021), *Penicillium* spp. (Li et al., 2019), nonpathogenic *Fusarium* spp. (Zhang et al., 2018b), *Bacillus* spp. (Luo et al., 2019), *Pseudomonas* spp. (Fatima and Anjum, 2017; Yu et al., 2017), and *Streptomyces* spp. (Abbasi et al., 2019; Heinsch et al., 2019; Peng et al., 2020), have been investigated to control Fusarium wilt. These BCAs employ a variety of strategies to control plant disease by both direct and indirect mechanisms. The most extensive studies on the mechanisms of biocontrol have focused on antibiosis. For example, *Streptomyces rimosus* M527 secretes the macrolides antibiotic, rimocidin, which inhibits fungal growth by interacting with cell membranes through ergosterol-forming channels (Song et al., 2020). A biocontrol strain of the Gram-positive bacterium *Bacillus amyloliquefaciens* SQR9 secretes the lipopeptide antibiotic bacillomycin D when SQR9 is confronted with *Fusarium oxysporum* (Li et al., 2014). In addition, the modes of action of disease suppression include competition for space, nutrients, or microelements, as well as degrading fungal virulence factors or priming of plant immunity (Chen et al., 2018).

Out of all BCAs, some species from the *Streptomyces* genus are regarded to be special in controlling plant disease because they exhibit many excellent traits. *Streptomyces* species are aerobic, Gram-positive, and spore-forming actinomycetes, which have a linear chromosome and several plasmids in a linear or circular form. They have relatively large genomes, approximately 8Mb to 10Mb in size depending on the specific species, and with high G+C content (69–73%; Hwang et al., 2014). One of the unique features of the genome of the *Streptomyces* species is the presence of biosynthetic gene clusters (BGCs) which encode enzymes contributing to the production of substantial secondary metabolites with multiple biological activities (Baltz, 2016). Genomic data have shown that *Streptomyces* spp. have the potential to produce even more secondary metabolites than have been isolated from them to date, as a large number of BGCs have been revealed (Lee et al., 2020). Consequently, the systematic investigation of *Streptomyces* species at the genetic level is becoming more important for counteracting pathogens. More importantly, their filaments and ability to sporulate help them cleave strongly to the rhizospheric soil particles forming a strong bond with the plants (Olanrewaju and Babalola, 2019). These traits are considered to be very useful in biological control of plant diseases.

In recent years, rapid development of high-throughput sequencing technology has provided new tools that are actively applied to study BCAs (Choi et al., 2015). Many BCAs are more deeply studied using genomics, and their metabolites are widely characterized using gas chromatography–mass spectrometry (GC–MS) and liquid chromatography-mass spectrometry technologies, which are providing new insights into their metabolism, biochemical properties, and potential for production of secondary metabolites (Pan et al., 2019). Previous study reported that a total of 21 different compounds identified from *Streptomyces* sp. SCA3-4 by GC–MS were composed of phenolic compound, pyrrolizidine, hydrocarbons, esters, and acids (Qi et al., 2019). *S. alfalfae* was originally described in 2016 and has already been used for fungal disease control (She et al., 2016). However, the mechanisms of *S. alfalfae* for promoting plant growth and inhibiting fungal pathogens remain to be elucidated. Elucidation of the biocontrol mechanisms of *S. alfalfae* at genomic level is necessary for the efficient application of such plant-beneficial microorganisms in agriculture.

In this study, we obtain a potential BCA, *S. alfalfae* XN-04, which displayed plant growth-promoting activity and a strong antagonistic activity against *Fov*. To better understand the mechanisms underlying the diverse and beneficial biological activities of this strain, we assessed the following objectives: (1) evaluate the biocontrol efficacy of XN-04 under greenhouse conditions, (2) evaluate the effect of XN-04 on cotton growth, (3) a comprehensive genome sequence analysis of XN-04, (4) characterize the metabolites produced by XN-04, and (5) evaluate the colonization ability of XN-04 in cotton roots, and all these information provided essential insights into the biocontrol properties of *S. alfalfae* XN-04.

## Materials and Methods

### Sample Collection and Isolation of Actinomycetes

The rhizosphere soil samples used in this study were collected in a cucumber commercial greenhouse (36°49′16″N, 101°36′43″E; Xining City, Qinghai Province, China) and a commercial cabbage field (33°24′26″N, 104°56′48″E; Longnan City, Gansu Province, China), respectively. The greenhouse has grown tomatoes and cucumbers over 5years and suffered from Fusarium wilt for more than 2years. The field has grown cabbage for more than 10years, and Fusarium wilt has occurred since 2009. A total of 10 healthy cucumber plants along with the rhizosphere soil (pH=7.6–7.8) were collected in May, 2016. Similarly, 10 healthy cabbage plants along with the rhizosphere soil (pH=7.5–7.6) were collected in June, 2016. The roots of plants were removed from the soil and the bulk soil was shaken off gently. All samples were placed in sterile plastic bags, subsequently transferred to the laboratory and air-dried for 3days.

Rhizosphere actinomycetes were isolated using dilution plating method (Awla et al., 2017). Briefly, the dried root samples (~ 1g rhizosphere soil attached) were suspended in 100ml sterile double-distilled water (ddH_2_O) with glass beads, incubated in an incubator shaker at 180 RPM at 28°C for 30min, and finally filtered with a double-layer gauze to obtain the rhizosphere soil suspension. Subsequently, 100μl of the dilution sample was placed on the surface of Gauze’s synthetic no. 1 (GS) medium that was supplemented with filter-sterilized potassium dichromate (5×10^−6^g/l). The plates were incubated at 28°C for 10days. Colonies of actinomycetes on the agar plates were picked on the basis of their growth rate, colony size, and morphological characteristics. Pure cultures were obtained by re-growth on GS medium and preserved as spore suspensions in 20% glycerol at −80°C freezer.

### Antifungal Activity Assay *in vitro*

A total of 13 phytopathogenic fungi were preserved in the Biological Control of Plant Disease Laboratory of Northwest Agriculture and Forestry University and were grown in Potato Dextrose Agar (PDA) plates at 28°C for 5days. All isolated actinomycetes were used for exploring antifungal activity as previously described with slightly modifications (Suarez-Moreno et al., 2019). Four wells (6mm diameter) were evenly perforated forming a cross around the center (25mm from center), and 100μl of each actinomycetes cell suspension (10^8^cfu/ml) was poured into the relative two wells. A negative control consisted of using the other two wells injected with the same amount of GS broth. A mycelial plug of 6mm diameter from the edge of every 5days old fungus was cut and transferred into the center of the actinomycetes-pregrown PDA plate. Petri dishes were incubated at 28°C for 7days, and the inhibition radius was measured. Each treatment had three plates, and the experiment was repeated three times.

### Evaluation of Biocontrol Efficiency

The biocontrol efficacy of XN-04 against *Fov* in a growth chamber was determined using the cotton cultivar Jimian 11 (susceptible to *Fov*) and the protocols described previously (Chen et al., 1995). Cotton seeds were surface sterilized with 4% sodium hypochlorite for 5min and rinsed three times in sterile distilled water (SDW). Culture pots (13cm high×11cm diameter) were filled with sterilized soil and 25ml of the different concentrations (10^6^∼10^9^cfu/ml) of XN-04 cell suspensions was added to the soil of each pot. Pathogen inoculation was performed 7days post-actinomycetes treatment by watering each plant with 25ml of the conidial suspension of *Fov* (10^8^ conidia/ml). A negative control was performed using uninoculated control seedlings treated with SDW only, while the positive control was inoculated with *Fov* and treated with SDW. In addition, carbendazim treatment was used as the chemical control.

All cotton plants were grown in a growth chamber at 23°C and 16h photoperiod, and watered with SDW every 3days. Two weeks after inoculation with *Fov*, all parameters were evaluated. Disease severity ratings were determined using a 0–4 rating scale (0, no symptoms; 1, 1–25% of leaves with symptoms; 2, 26–50% of leaves with symptoms; 3, 51–75% of leaves with symptoms; and 4, 76–100% of leaves with symptoms). Each treatment had 15 cotton plants, and the experiment was repeated three times.

Disease index and biocontrol efficiency were calculated as follows:


Disease index=∑number of diseased plants of each grade×value of relative gradetotal number of investigated plants×4×100

$$\mathrm{Control efficiency}=\frac{\begin{array}{l} \mathrm{disease index of control group}-\\ \mathrm{disease index of treatment group} \end{array}}{\mathrm{disease index of control group}}\times 100\%$$

### Plant Growth-Promoting Experiments

In each treatment, a total of 100 cotton seeds were fully submerged in 100ml XN-04 cell suspensions (10^6^∼10^9^ cfu/ml) for 12h. The treated seeds were placed into a plug tray (53×27×4cm deep) filled with sterilized soil, with one seedling occupying each cell. The seeds submerged in SDW were used as the negative control. The biometric properties (plant height, root length, fresh weight, and dry weight) were measured at the 20th days after seed sowing. This experiment was repeated three times.

### Genome Sequencing, Assembly, Annotation, and Bioinformatics Analysis

#### DNA Extraction

The genomic DNA of XN-04 was extracted using a Wizard® Genomic DNA Purification Kit (Promega) according to the manufacturer’s protocol. Purified genomic DNA was quantified by the TBS-380 fluorometer (Turner BioSystems, Sunnyvale, CA). High-quality DNA (OD260/280=1.8~2.0, > 20μg) was used to conduct the further research.

#### Sequencing, Assembly, and Annotation

The whole genome of XN-04 was sequenced using a combined strategy of Illumina PE platform and PacBio RS II Single Molecule Real Time (Majorbio Bio-pharm Technology Co., Ltd., Shanghai, China). The reads were *de novo* assembled using both canu version 1.4 (Koren et al., 2017) and SPAdes version 3.8.2 (Bankevich et al., 2012). The protein-coding genes (CDSs) prediction was carried out using Glimmer version 3.02 (Delcher et al., 2007). The rRNA and tRNA were analyzed using Barrnap version 0.8 (Seemann and Booth, 2018) and tRNA-scan-SE version 2.0 (Lowe and Eddy, 1997), respectively. Gene annotation was carried out in BLAST searches of widely used databases, i.e., NCBI non-redundant (NR), Swiss-Prot (Apweiler et al., 2004), Pfam (Finn et al., 2014), Clusters of Orthologous Groups (COG; Jensen et al., 2008), Gene Ontology (GO; Ashburner et al., 2000), and Kyoto Encyclopedia of Genes and Genomes (KEGG; Kanehisa and Goto, 2000) databases. The circular graphical representation of chromosome and plasmid was analyzed using a comparative genomics tool CGView 2.0 (Stothard and Wishart, 2005). Carbohydrate-active enzymes were annotated according to the Carbohydrate-Active enZYmes (CAZy) database using the dbCAN2 meta server (Zhang et al., 2018a). In addition, the antibiotic and secondary metabolite production gene clusters were examined using the program antiSMASH version 6.0 (Blin et al., 2021).

### Identification and Characterizations of XN-04

#### Phylogenetic Analyses

The 16S rRNA gene sequences were used as queries in BLAST searches through the NCBI GenBank nucleotide database. Multiple alignments of 16S rRNA gene sequences and 31 house-keeping gene sequences were conducted using the program Clustal X, respectively (Larkin et al., 2007). The phylogenic tree was constructed using the neighbor-joining method with the program MEGA version 7.0 (Center for Evolutionary Functional Genomics, United States; Kumar et al., 2016).

#### Cultural and Morphological Characterizations

Morphological characteristics of XN-04, including spore size and surface ornamentation, were observed by scanning electron microscopy (SEM; Hitachi model S-3400N, Japan) of 14day old cultures grown on MS medium. The cultural characteristics of XN-04, including color of spore mass, aerial mycelium, substrate mycelium, and diffusible pigments, were recorded after incubation at 28°C for 14days on ISP, PDA, GS, and mannitol soybean (MS) medium, respectively.

#### Strain Characterizations

The utilization of sole carbon and nitrogen sources was tested as previously described (Williams et al., 1983). Other biochemical characteristics tests were carried out according to *Bergey’s Manual of Systematic Bacteriology* (Holt et al., 1994). The temperature sensitivity for growth was determined from 5°C to 50°C at intervals of 5°C on Bennett medium after incubation for 7days. The NaCl tolerance [0–10% (M/V) in 1% intervals] was evaluated after XN-04 growth on Bennett medium at 28°C for 7days. In addition, XN-04 was characterized for its ability to produce extracellular enzymes (i.e., chitinase, cellulolytic, β-1, 3-glucanase, protease, and lipase), indoleacetic acid (IAA), 1-aminocyclopropane-1-carboxylic acid (ACC) deaminase, siderophores, HCN, and phosphate solubilization as described elsewhere (Jang and Chen, 2003; Nascimento et al., 2020).

### Antifungal Activity of XN-04 Extract

#### Production and Extraction of Antifungal Metabolites

The strain XN-04 was cultured in GS broth at 180 RPM at 28°C for 14days. A total of 10l fermentation broth was centrifuged at 5000 RPM at 4°C for 10min to pellet cells and to generate a cell-free supernatant. The cell-free supernatant was evaporated to dryness using a rotary vacuum evaporator under reduced pressure at 50°C. The mycelia were extracted with methanol (MeOH) and then concentrated *in vacuo*. Subsequently, four solvents of different polarity, i.e., petroleum ether (PE), dichloromethane (DCM), ethyl acetate (EtOAc), and n-butanol, were used to extract antifungal metabolites from the MeOH extract, respectively. All organic solvent extracts were concentrated to 50mg/ml. Antifungal activity was evaluated with concentrations of 50μg/ml using the method as described by Mei et al. (2019).

#### TLC Analysis

The EtOAc extract (10mg/ml) was analyzed by Thin layer chromatography (TLC) using DCM: MeOH (6:1, v/v) as solvent system. The developed TLC plates were air-dried to remove all traces of solvents and visualized under ultra-violet (UV) at 254nm (absorbance) and stained with iodine. To further analyze the number of active antifungal metabolites in the EtOAc extract, TLC chromatogram strips were placed on the surface of PDA plates containing *Fov* spores. Plates were incubated at 28°C for 3days. The number of antifungal metabolites in the EtOAc extract was determined by observing clear zones using Retardation factor (Rf) values.

#### Stability of Antifungal Metabolites

To examine the effect of temperature, the EtOAc extract was incubated at 20, 40, 60, 80, 100, and 121°C for 1h, respectively. After the sample was cooled to room temperature, the antifungal activity was tested. For the pH test, HCl and NaOH solutions were used to adjust the EtOAc extract pH from 1.0 to 10.0 at intervals of 1.0. Afterward, the pH values were readjusted to 7.0, and the residual antifungal activity was tested. For the proteinase *K* test, EtOAc extract was treated with 0.1mg/ml of proteinase K at 37°C for 60min. Also, the EtOAc extract was exposed to sunlight for 12–96h and exposed to UV light for 0–4h to check photostability, respectively. The antifungal activities were evaluated using the method described above. All experiments were performed in triplicate.

### GC–MS Analysis

GC–MS was used to identify the chemical compounds in the extract of XN-04. Briefly, GC–MS was performed on a Shimadzu GC-2030 with a triple quadrupole mass spectrometer (TQ-8050 NX). Helium as a carrier gas was injected at 1ml/min. The column temperature was programmed initially at 60°C for 1min, increased to 100°C at 5°C/min for 5min, raised at 10°C/min to 250°C for 35min, and finally kept to 280°C at 10°C/min for 25min. The mass spectrometer was operated in electron ionization (EI) mode at 70eV, with an interface temperature of 280°C, an ion source temperature of 240°C, a mass spectrometer acquisition delay time of 3.5min, and a continuous scan from 50 to 650amu. Peaks were identified in comparison with the mass spectra data against the National Institute of the Standards and Technology (NIST) spectral library.

### Transformation and Colonization of XN-04

#### Creation of the Strain XN-04 Labeled With EGFP

*Escherichia coli* strain ET12567 (harboring the helper plasmid pUZ8002) and plasmid pIJ8641 (carrying the EGFP gene under the constitutive *ermE* promoter) were preserved in the Biological Control of Plant Disease Laboratory. Plasmid pIJ8641 was transformed into the donor strain *E. coli* ET12567 (pUZ8002) and then conjugated into recipient XN-04 as previously described (Kieser et al., 2000; Zhang et al., 2021). Genomic DNA of wild-type (WT-) and transformed (t-) XN-04 was extracted, and the quality was tested by primers 27F (5′-AGAGT TTGAT CCTGG CTCAG-3′) and 1492R (5′-GGTTA CCTTG TTACG ACTT-3′; Huang et al., 2020). The EGFP gene was detected in the t-XN-04 strain following the protocol described before (Bonaldi et al., 2015) and the expression was observed by Confocal Laser Scanning Microscope (CLSM; Olympus model FV3000, Japan). The t-XN-04 growth rate, mycelium morphology, and inhibiting effect against *Fov* were examined and compared to WT-XN-04.

#### Colonization of Cotton Seedlings by EGFP-Labeled XN-04

Cotton seeds (Jimian 11) were surface sterilized as described above. Surface-sterilized seeds were pre-germinated on filter paper at 28°C for 2days. The germinated seeds were placed into plug tray (53×27×4cm deep) filled with sterilized soil, with one seeds occupying each cell. All cotton seedlings incubated in a growth chamber at 23°C with a 16h photoperiod. After 7days of incubation, 1ml EGFP-labeled XN-04 cell suspensions (10^8^cfu/ml) were uniformly distributed in every cell. The seedlings treated with SDW were used as negative control.

The colonization ability of EGFP-labeled XN-04 in the cotton rhizosphere was quantified and observed at 2, 4, and 7days after inoculation. In general, cotton seedlings were carefully removed from the soil, and large soil aggregates were removed by gently shaking the plants. For plating count assay, the complete root parts were suspended in 10ml sterile ddH_2_O and vortexed for 10min. Then, the suspension was serially diluted with sterile ddH_2_O, and 100μl cell suspension from various dilutions (10^−2^, 10^−3^, or 10^−4^) was plated into GS plates supplemented with apramycin (50μg/ml). Plates were incubated at 28°C for 7days, and the number of colony forming unites was determined. The colonization density of the EGFP-labeled XN-04 in rhizosphere was expressed as cfu/g of roots weight. The experiment was repeated three times with 10 root samples per replicate.

For CLSM observation, the roots were cut into 2cm lengths, placed on microscope slides, and visualized using CLSM at an excitation wavelength of 488nm. All experiments were repeated using 10 roots, and representative results are shown.

### Statistical Analysis

Statistical analysis, one-way ANOVA, and Duncan’s multiple range test were performed using the software SPSS 24.0 (Chicago, IL, United States) in this study.

## Results

### Antagonistic Activity of XN-04 Against *Fov in vitro* and in Planta

**Figure 1:**
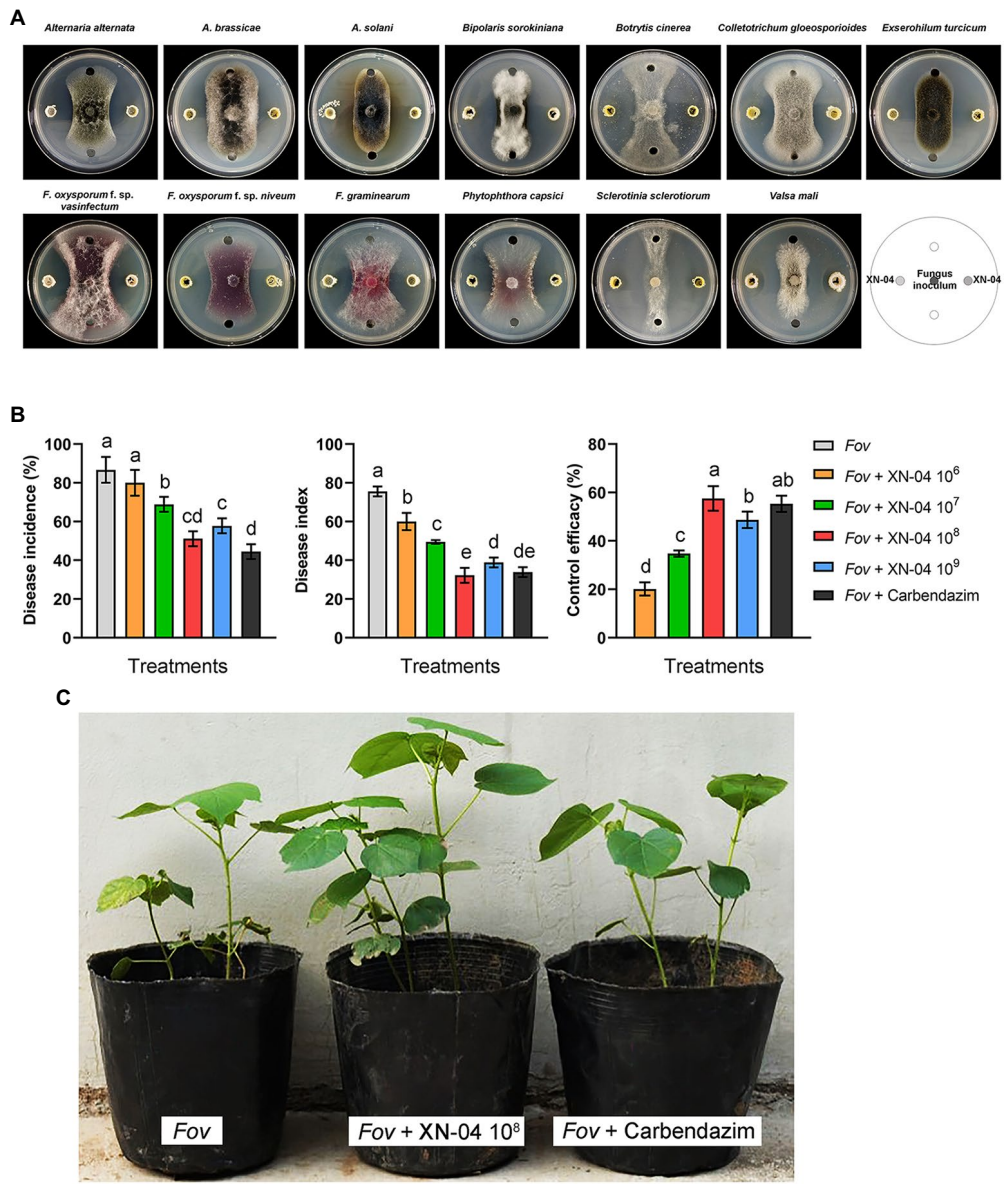
The antifungal activity of XN-04. **(A)** Antagonistic activity of XN-04 against 13 important phytopathogenic fungi; **(B)** biocontrol efficacy of XN-04 against *Fov* under greenhouse conditions; and **(C)** the symptoms of Fusarium wilt disease development on cotton in each treatment in greenhouse experiment. Data are mean±SD (*n*=5). Different lowercase letters indicate a significant difference at *p*<0.05 level by Duncan’s new multiple range test.

A total of 32 morphologically different actinomycetes (numbered GS-01 to GS-10 and XN-01 to XN-22) were isolated from the rhizosphere soil collected in Gansu and Qinghai Provinces, China. Among them, 16 actinomycetes exhibited various degrees of inhibitory activities against *Fov* (Supplementary Table S1). Notably, an actinomycete isolate (termed XN-04) demonstrated strong inhibitory activity against *Fov* during co-cultivation, producing a radius of inhibition zone >13mm, and an inhibition zone >10mm against several other fungal pathogens (Figure 1A). To determine whether XN-04 inhibited the growth of *Fov* in planta, we further conducted biocontrol experiments under greenhouse settings. Similar to Carbendazim^®^, a fungicide widely used to control *Fov* in China, treatment with XN-04 at the optimum concentration (10^8^cfu/ml) by soil inoculation significantly (*p*<0.05) reduced the wilt incidence and disease index (Figures 1B,C). These results indicate that XN-04 is an effective BCA for the control of Fusarium wilt of cotton.

### Plant Growth-Promoting Activity of XN-04

**Figure 2:**
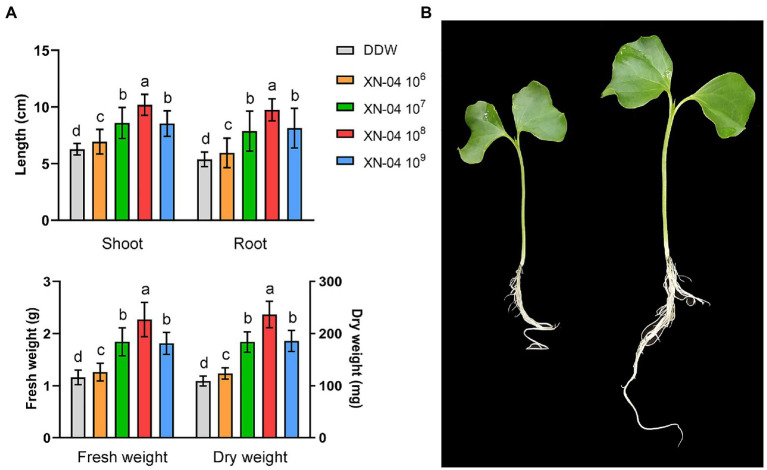
Cotton plants growth-promoting assay. **(A)** Data for shoot length, root length, fresh weight, and dry weight of cotton plants 14days after XN-04 inoculation. Data are mean±SD (*n*=5). Different lowercase letters indicate a significant difference at *p*<0.05 level by Duncan’s new multiple range test. **(B)** Overall plant development.

To determine the effect of XN-04 on the growth of cotton seedlings, plants were harvested and all plant growth parameters were assessed at the 20th day after seed sowing. As shown in Figure 2A, the plant growth-promoting effect increased with the increased concentration of XN-04 and then declined at a high concentration. The optimal effect was observed at a concentration of 10^8^cfu/ml. When compared to the non-inoculated control, values of shoot length, root length, fresh weight, and dry weight of the cotton seedlings inoculated with XN-04 cell suspension (at 10^8^cfu/ml) were increased by 62.52, 81.56, 95.69, and 117.43%, respectively (Figures 2A,B).

### Identification and Characteristics of XN-04

**Figure 3:**
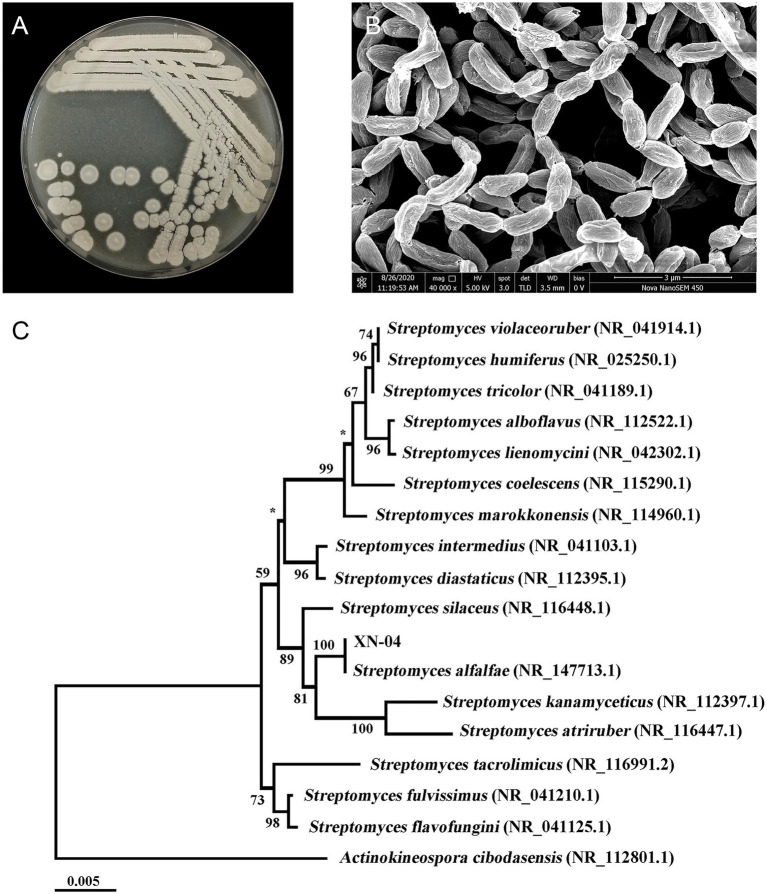
Identification of XN-04. **(A)** Colony morphology of XN-04 on MS medium after 14days of incubation at 28°C. **(B)** SEM of XN-04 grown on MS medium for 14days at 28°C. **(C)** A phylogenetic tree based on 16S rRNA gene sequences showing the phylogenetic position of XN-04 and the type strains of related taxa. The branches are scaled in terms of the expected number of substitutions per site (see bar). Support values from neighbor-joining bootstrapping are shown above the branches if >50%. Bar, 0.005 expected substitutions per site.

To characterize XN-04, the isolate was grown in the standard medium described in the International *Streptomyces* Project (ISP) and micromorphology of sporulation was examined by SEM. XN-04 exhibited good growth on ISP 3, MS and PDA medium, and moderate growth on the other tested organic and synthetic medium. Moreover, XN-04 did not produce any water-diffusible pigments in any tested medium. For sporulation, different spore masses were yielded on different agar plates, with the largest number of spores obtained on MS medium followed by the spore yields on ISP 3 and PDA medium, sequentially. The detailed cultural characteristics of XN-04 are shown in Supplementary Table S2. The colony morphology of XN-04 showed a hard surface with white aerial mycelia and light pink spores (Figure 3A). Morphological observation of the 2weeks culture of XN-04 grown on MS medium revealed that it had typical morphological properties of the genus *Streptomyces*. Aerial and substrate mycelia were well developed without fragmentation. Sporulation in XN-04 featured cylindrical spores forming a flexuous spore chain with a rough surface (Figure 3B). Biochemical tests indicated that XN-04 was able to utilize almost all the tested carbon and nitrogen sources except for mannitol. XN-04 was found to grow at a temperature range of 5 to 35°C and NaCl tolerance of 0 to 8% (optimum NaCl of 1 to 2%). The other physiological and biochemical properties of XN-04 are given in Supplementary Table S3.

The XN-04 16S ribosomal RNA genes have six copies. A BLAST analysis of the complete 16S rRNA gene sequences revealed that XN-04 was closely related to *S. alfalfae* ACCC40021 with 100% similarity. Next, phylogenomic trees were constructed based on the 16S rRNA gene and 31 house-keeping genes sequences using the neighbor-joining methods, respectively. XN-04 was grouped together with *S. alfalfae* ACCC40021 with a high bootstrap value (100; Figure 3C and Supplementary Figure 1). Altogether, XN-04 is recognized as a new member of the *S. alfalfae* species.

### Genome Features of XN-04

**Figure 4:**
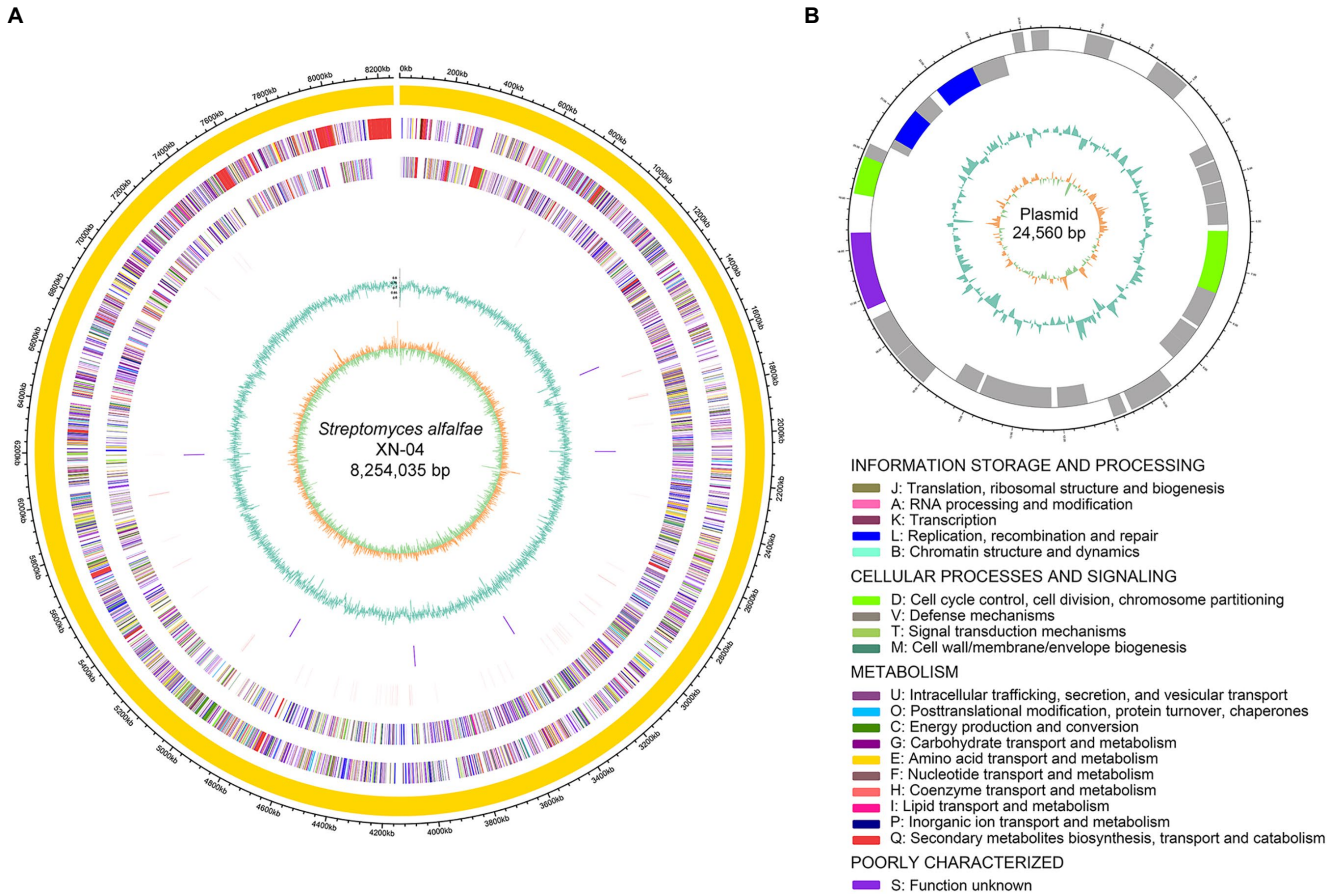
Genome map of XN-04. **(A)** Genetic map of the circular chromosome; **(B)** genetic map of the circular plasmid. The circular map consists of six circles, from the outside circle to the inward. Circle 1 shows the distribution of genes related to COG categories in the forward strand; circle 2 shows CDS region including tRNA, rRNA, and others in forward strand; circle 3 shows CDS region including tRNA, rRNA, and others in the backward strand; circle 4 shows the distribution of genes related to COG categories in the backward strand; circle 5 shows the GC content; and circle 6 shows the GC skew.

In order to fully understand the molecular mechanisms of plant growth-promoting and antagonism, the genome of XN-04 was sequenced. The complete genome of XN-04 consists of an 8,254,035bp linear chromosome (Figure 4A) and a 24,560bp circular plasmid (Figure 4B), with average GC content of 72.06% (Table 1). The complete chromosome and plasmid sequences for XN-04 have been deposited in the GenBank database with accession numbers CP060742 and CP060743, respectively. Analysis of the genome revealed that it contained 7,579 CDSs which account for approximately 88.36% of the genome.

**Table 1:** Genome features of *Streptomyces alfalfae* XN-04.

Features	Chromosome	Plasmid	Genome
Genome topology	Linear	Circular	–
Assembly size (bp)	8,254,035bp	24,560bp	8,278,595bp
G+C content (%)	72.06	71.85	72.06
Protein-coding genes (CDSs)	7, 549	30	7, 579
tRNA genes	72	0	72
rRNA genes	18	0	18
Gene total length (bp)	7,297,671bp	17,151bp	7,314,822bp
Secondary metabolite gene clusters	33	0	33
GenBank accession	CP060742	CP060743	–

Functional analysis by COG, GO, and KEGG revealed that 5,657, 4,976, and 2,726 out of the 7,579 identified CDSs were assigned to COG, GO, and KEGG categories, respectively. These 7,579 CDSs could be assigned to four broadly functional categories by COG analysis. The predicted proteins associated with metabolism were the most abundant type (40.78%), followed by poorly characterized (38.18%), information storage and processing (19.53%), and cellular processes and signaling (16.76%; Supplementary Figure 2). To explain the relevance of the genome of XN-04, GO analysis was used to categorize genes into three categories according to matching with known sequences. In three categories, molecular function contained the most numerous GO terms and gene number (3,990), followed by biological process (3,449) and cellular component (1,949; Supplementary Figure 3). In KEGG metabolism annotations of XN-04, of the six classifications of KEGG pathways, metabolism contained the most number of genes, followed by environmental information processing (Supplementary Figure 4). Additionally, the results showed that 285 genes were identified from CAZy families and are distributed into five classes. A total of 108 proteins were predicted as belonging to the Glycoside Hydrolase (GH) family, 66 to Glycosyl Transferases (GTs), 63 to Carbohydrate Esterases (CEs), 22 to Auxiliary Activities (AAs), 20 to Carbohydrate-Binding Modules, and 6 to the Polysaccharide Lyases (PLs) family (Supplementary Figure 5).

### Genome Analysis of Secondary Metabolite Clusters

Genome mining of the XN-04 genome revealed that this strain has the potential to produce a wealth of secondary metabolites. Up to 34 putative gene clusters for secondary metabolite biosynthesis located to the 8.25Mb chromosome of XN-04 as determined by antiSMASH 6.0 online software (Table 2). Among these clusters, three encode non-ribosomal peptides (NRPS, including biosynthesis mirubactin, streptolydigin, and coelichelin); one encodes type I polyketide synthases (PKS I, including biosynthesis ebelactone); two encode PKS III (including biosynthesis alkylresorcinol and violapyrone B); four are responsible for hybrid peptide polyketide; and five appear to code for terpene (Table 2). The remaining 19 gene clusters are mainly involved in the biosynthesis of ectoine, siderophore, bacteriocin, etc. Notably, XN-04 shows high similarity (100%) with seven known clusters.

**Table 2:** Secondary metabolite clusters in *S. alfalfae* XN-04.

Cluster	Type	Most similar known cluster	Similarity	Gene no.	Length (bp)
1	NRPS, T1PKS, PKS-like, butyrolactone	microtermolide A	46%	65	98,331
2	NRPS, RiPP-like	triacsins	6%	39	57,759
3	lassopeptide	moomysin	75%	21	22,512
4	NRPS, RiPP-like	chalcomycin A	9%	34	76,982
5	T3PKS	alkylresorcinol	100%	33	40,333
6	CDPS	-	-	18	20,831
7	NRPS-like, T1PKS	borrelidin	20%	43	43,720
8	T1PKS, other, terpene, RiPP-like, NRPS, indole	A-503083 A/A-503083 B/A-503083 E/A-503083\u00B0F	7%	99	113,097
9	NRPS, T1PKS	GE81112	10%	42	57,369
10	terpene	isorenieratene	100%	27	23,986
11	T3PKS	violapyrone B	28%	40	38,709
12	NRPS	mirubactin	50%	33	48,703
13	ectoine	ectoine	100%	9	10,405
14	NRPS, T1PKS	GE81112	7%	42	49,396
15	prodigiosin	undecylprodigiosin	59%	25	34,520
16	melanin	istamycin	5%	11	10,438
17	thioamide-NRP, NRPS, ladderane	ishigamide	100%	52	77,897
18	other, T1PKS, butyrolactone	chlorizidine A	11%	46	53,063
19	terpene	albaflavenone	100%	17	19,462
20	NRPS	streptolydigin	5%	43	55,724
21	siderophore	ficellomycin	3%	9	15,029
22	NRPS	coelichelin	100%	30	50,594
23	terpene	pentalenolactone	58%	15	19,524
24	RiPP-like	–	–	11	11,284
25	terpene	geosmin	100%	12	19,626
26	lanthipeptide-class-v‚ T1PKS	calicheamicin	6%	54	61,926
27	NRPS-like	maklamicin	4%	47	61,526
28	terpene	hopene	92%	22	26,622
29	T3PKS, NRPS	kistamicin A	64%	44	88,158
30	NRPS, T1PKS, indole, transAT-PKS-like	5-isoprenylindole-3-carboxylate β-D-glycosyl ester	28%	49	68,398
31	NRPS, betalactone, T3PKS	streptolydigin	10%	59	67,672
32	NRPS-like	hygromycin A	12%	39	45,786
33	NRPS, transAT-PKS	oxalomycin B	87%	42	103,768
34	T1PKS	ebelactone	13%	25	125,246

### Genes Associated With Fungal Cell Wall Degrading Enzymes

XN-04 contains various genes encoding specific enzymes involved in the degradation of chitin, including eight chitinases (seven from the GH18 family and one from the GH19 family), six β-N-acetyl hexosaminidase (three from the GH3 family and three from the GH20 family), and one chitosanase from the GH46 family. In addition, XN-04 also has six chitin-binding protein from the AA10 family, which enhance the binding abilities of enzymes to insoluble substrates. Details of the enzymes are listed in Supplementary Table S4. For the degradation of glucan, XN-04 harbors four genes encoding endo-1, 3-β-glucanase (three from the GH16 family and one from the GH64 family; Table S4). Moreover, XN-04 contains a variety of genes encoding enzymes that are thought to be involved in the degradation of cellulose, protein, and lipids (Supplementary Tables S4, S5).

### Genes Associated With Plant Growth Promotion

Bioinformatics analysis of the XN-04 genome showed the presence of several genes contributing directly or indirectly to plant growth-promoting activities.

#### Plant Hormones

Three trp-dependent indole-3-acetic acid biosynthesis pathways, the indole acetamide (IAM), indole-acetonitrile (IAN), and the tryptamine (TAM) pathways appear to be involved in the IAA biosynthetic capacity of XN-04. In the IAM pathway, tryptophan can be directly converted to IAM by a tryptophan monooxygenase enzyme. Then IAM is converted to IAA by the action of the amidase enzyme. Six tryptophan 2-monooxygenase encoding genes and three amidase encoding genes are found in the XN-04 chromosome (Supplementary Table S6). In the TAM pathway, tryptophan can be converted to TAM, which is subsequently converted to indole-3-acetaldehyde (IAAld) by an amine oxidase. Then, the IAAld is converted to IAA by the action of an aldehyde dehydrogenase enzyme. Two monoamine oxidase encoding genes and four aldehyde dehydrogenase are present in the XN-04 chromosome (Supplementary Table S6). In addition, XN-04 also contains a nitrilase encoding gene, which is involved in the process of transforming IAN to IAA *via* the IAN pathway (Supplementary Table S5). The presence of three separate IAA biosynthesis pathways in XN-04 indicates that IAA production plays an important role in XN-04 plant growth-promoting activities.

Additionally, XN-04 also contains a putative ACC deaminase that can decompose ACC (Supplementary Table S6) and thus inhibit ethylene synthesis in plants to improve the ability of plants to survive under adverse conditions and to provide adaptability to the environment.

#### Siderophore Biosynthesis and Transport

The XN-04 genome contains abundant genetic elements involved in siderophore biosynthesis and iron complex transport (Supplementary Table S7). Moreover, three siderophore biosynthesis clusters (cluster 12, 19, and 20) are also present in the chromosome sequence of XN-04 (Table 2). Notably, cluster 22 shows 100% similarity to the biosynthetic gene cluster of coelichelin in *S. coelicolor* A3(2), including a siderophore biosynthesis protein gene (gene6002) and eight genes related to iron transport (gene 5,989, gene5997, gene5998, gene5999, gene6000, gene6001, gene6003, and gene6014).

#### Phosphate Solubilization

Strain XN-04 genomic DNA contains two *ppx* genes encoding exopolyphosphatase, and a *ppa* gene encoding for inorganic pyrophosphatase, that are involved in the degradation of inorganic polyphosphates. The strain XN-04 genome also contains four *phoD* genes, encoding alkaline phosphatase, which are involved in organic phosphate solubilization. Furthermore, one *pstABCS* phosphate transport system is found in the chromosomal DNA and involved in the transport and degradation of phosphonates (Supplementary Table S8).

### Enzymes and Secondary Metabolites Production

*In vitro* tests suggested that XN-04 could grow well on colloidal chitin, glucan, sodium carboxymethyl cellulose, casein, and Rhodamine B medium, with a clear zone surrounding the colonies as a result of substrate hydrolysis indicating that XN-04 could secrete chitinase, β-1, 3 glucanase, cellulose, protease, and lipase (Table 3). Moreover, XN-04 showed the ability to decolor the blue-colored ferric CAS complex into orange, revealing the production of siderophore. In addition, XN-04 was able to solubilize organic phosphate and showed a remarkable ability to produce plant hormones IAA and ACC deaminase (Table 3).

**Table 3:** Production of enzymes and secondary metabolites by *S. alfalfae* XN-04.

Hydrolytic activity	Trait	Plant growth-promoting ability	Trait
Chitinase	+	Siderophore	+
β-1, 3 glucanase	+	Indole Acetic Acid (IAA)	+
Cellulase	+	Hydrogen cyanide (HCN)	−
Protease	+	ACC deaminase	+
Lipase	+	Organophosphate solubilization	+
		Inorganic phosphate solubilization	−

“+” *Positive reaction;* “−” *Negative reaction*.

### Property of XN-04 Active Substances

**Figure 5:**
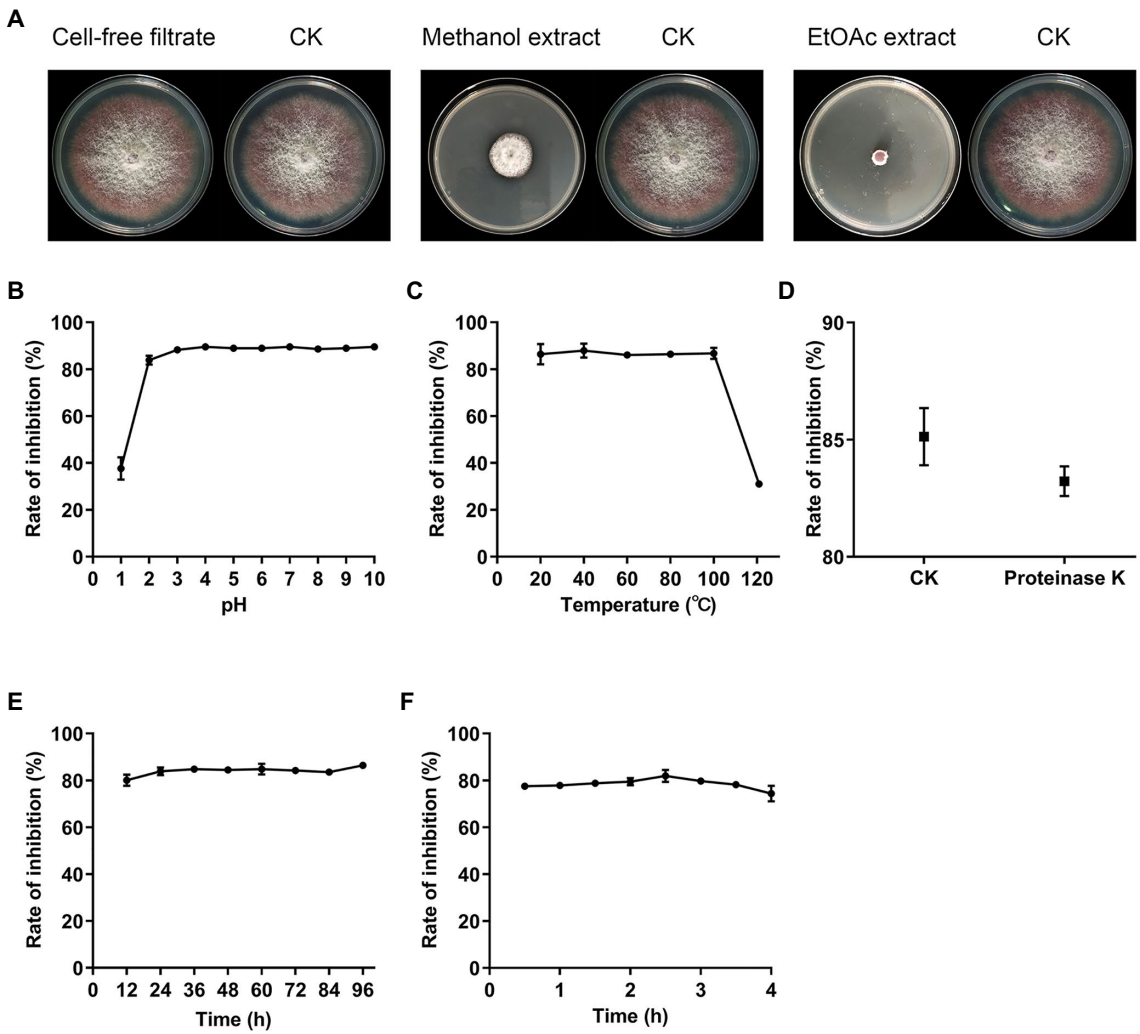
The distribution and stability of active substances. **(A)** Antagonistic activities against *Fov* of secondary metabolites extracted from XN-04. **(B)** The effect of temperature **(C)** pH **(D)** proteinase K **(E)** lighting, and **(F)** UV on XN-04 active substances. Data are mean±SD (*n*=3).

For the production of antifungal metabolites, fermentation was performed in GS broth for 14days. After fermentation, MeOH extract of mycelia showed obvious antifungal activity against *Fov*, while the cell-free filtrates of XN-04 exhibited no inhibitory effects on the growth of *Fov* (Figure 5A). Further extraction revealed that all XN-04 antifungal metabolites were concentrated in EtOAc fractions (Figure 5A). TLC using DCM: MeOH (6:1, v/v) as solvent system resulted in separation of five antifungal metabolites in the EtOAc extract with Rf values of 0.93, 0.88, 0.33, 0.25, and 0.15 (Supplementary Figure 6).

The stability of these antifungal metabolites presented in XN-04 to various physical and biochemical stresses was then examined to show the application performance of XN-04 and its metabolites. According to the acid–base stability assay, the antifungal activity of EtOAc extract declined markedly after exposure to acidic conditions at a pH of 2 or lower. However, the antifungal activity showed no significant difference at a pH within the range of 2 to 10 (Figure 5B). The antifungal activity remained unchanged after being kept at 100°C for 1h but decreased significantly at 121°C (Figure 5C). Furthermore, these metabolites were found to be stable when treated with light, UV, and proteinase K (Figures 5D–F). Therefore, it is undeniable that XN-04 was stable under most conditions, which might indicate its biotechnological potential in fields under varying climatic conditions and in different regions.

### GC–MS Analysis

From the GC–MS analysis, 29 chemical compounds of the MeOH extract of XN-04 were identified by the NIST library based on their retention time, molecular mass, and molecular formula (Supplementary Table S9). Among these compounds (1) n-hexadecanoic acid, (2) 9,12-octadecadienoic acid (Z,Z)-, methyl ester, (3) 9,12-octadecadienoic acid (Z,Z)-, (4) 9-octadecenoic acid (Z)-, and (5) bis(2-ethylhexyl) phthalate were reported with antifungal activity (Walters et al., 2004; Rahman and Anwar, 2006; Pinto et al., 2017; Qi et al., 2019).

#### Colonization of XN-04 in Cotton Roots

**Figure 6:**
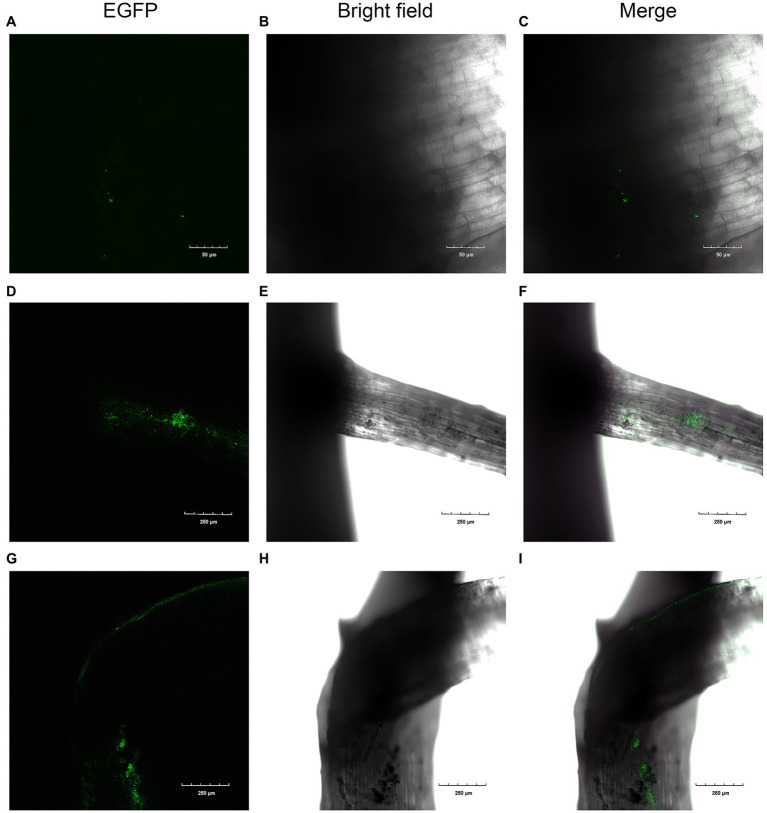
Colonization of cotton roots by EGFP-labeled XN-04. Colonization after **(A**, **B**, and **C)** 2days; **(D**, **E**, and **F)** 4days; and **(G**, **H**, and **I)** 7days.

To confirm whether XN-04 colonized cotton roots, a plasmid pIJ8641 containing the enhanced green fluorescent protein (EGFP) gene was transfected into XN-04 to construct an EGFP-labeled XN-04. The EGFP gene was detected in XN-04 and its expression was confirmed by CLSM observation. After transformation, the growth rate, mycelium morphology, and inhibiting effect on *Fov* were detected and they are similar to the wild-type XN-04 (data not shown). At the 2th, 4th, and 7th day after inoculation, cotton seedling roots were observed by CLSM. No XN-04 cells were observed in the uninoculated cotton seedlings (data not shown). Two days after inoculation, a few EGFP-labeled XN-04 was found adhered to the surface of the cotton lateral roots (Figures 6A–C). At the 4th day after inoculation, XN-04 cells were found to have colonized the surfaces of both the primary and lateral roots in the maturation zone (Figures 6D–F). CLSM results at the 7th day after inoculation revealed that the number of EGFP-labeled XN-04 accumulated on cotton roots had increased (Figures 6G–I).

The colonization density of the EGFP-labeled XN-04 in the cotton rhizosphere was further quantified using the traditional plating count method. With increasing time following inoculation, the number of EGFP-labeled XN-04 was found to have increased in cotton rhizosphere. The highest number of bacteria was reached on the 7th day post-inoculation at 1.33×10^6^cfu/g root weight (Supplementary Table S10).

## Discussion

A considerable number of actinomycetes, especially *Streptomyces* spp., have been selected as BCAs promising resources for the biocontrol of fungi-induced plant diseases (Cho et al., 2017; Fidan et al., 2019). Since biocontrol agent selection is not a simple task due to the complexity of the field environment, BCAs with multiple modes of action and functions should be selected for further studies rather than selecting a BCA with only one mode of action. In this study, *S. alfalfae* XN-04 was isolated from rhizosphere soil, which can colonize the cotton plant root system, inhibiting the mycelial growth of *Fov* by production of hydrolytic enzymes and antifungal secondary metabolites. More importantly, XN-04 is a versatile plant growth-promoting streptomycete, apparently modulating plant growth and development. Considering its above excellent traits, strain XN-04 may have great potential to be developed as a novel BCA for Fusarium wilt in future.

### The Colonization Ability of XN-04

Effective colonization on plant roots is crucial for the biocontrol of soil-borne plant diseases by BCA (Ye et al., 2020). Before a species can be selected as a BCA, the level of root colonization must be determined. It must be able to compete favorably against other microbes to be able to exert its influence on the host plants. Coombs and Franco (2003) demonstrated the endophytic colonization of GFP-labeled *Streptomyces* sp. EN27 was observed from a very early stage of plant development with colonization of the embryo, endosperm, and emerging radicle of wheat. Chen et al. (2016) reported that the abundant colonization of young lettuce seedlings by the EGFP-labeled *S. exfoliatus* FT05W demonstrated *Streptomyces*’ capability to interact with the host from early stages of seed germination and root development. In this study, CLSM observation indicated that XN-04 began to colonize the roots of young cotton seedlings at 2days after inoculation (Figure 6). The strain XN-04 quantity in cotton root surrounding soil reached a level of 1.33×10^6^cfu/g root at 7days after inoculation (Supplementary Table S10). This finding suggests that XN-04 was well adapted to the rhizosphere soil environment.

Biochemical tests revealed that XN-04 is able to use a rather wide range of compounds as carbon and nitrogen sources, which confer a significant advantage to the plant colonization ability. Additionally, XN-04 exhibited a good tolerance to salt as it grew in the presence of 0–8% (w/v) NaCl. In the study, an ectoine biosynthesis cluster was found in the XN-04 chromosome (Table 3). Ectoine is one of the most important synthetic osmotic compatible solutes for moderate halophilic bacteria, which can also equilibrate the osmotic pressure of cells, as well as provide a reversible assistance to enzymes, DNA, and the whole cell in adverse environments (Prabhu et al., 2004). All of these properties suggest that *S. alfalfae* XN-04 may have the ability to adapt to the complex field soil conditions.

### Antifungal Metabolites Analysis of XN-04

Highly active hydrolytic enzymes including chitinases, β-1, 3 glucanase, and protease, produced by *Streptomyces* spp., may confer capability for competing in the rhizosphere environment, destroying the cell walls of phytopathogenic fungi, and making these microorganisms promising for biocontrol use (Vurukonda et al., 2018). Since chitin is the most abundant component of the fungal cell wall, chitinases have been widely demonstrated to be important inhibitors of fungal growth (Taechowisan et al., 2003). A recent study showed that the GH19 chitinase cloned from *S. alfalfae* ACCC40021 exhibited great capability for inhibiting the growth of phytopathogenic fungi (Lv et al., 2021). In the present study, XN-04 exhibited the ability to degrade chitin on agar plates, which was also confirmed by the identification of the genes related to chitin degradation in its genome. Therefore, we hypothesize that the excellent biocontrol property of XN-04 may be related to chitinase produced by XN-04.

Apart from hydrolytic enzymes, the function of bioactive secondary metabolites produced by *Streptomyces* spp. cannot be underestimated for controlling fungal diseases (Olanrewaju and Babalola, 2019). In this study, antiSMASH analysis revealed that a total of 34 secondary metabolites BGCs are present in the genome of the XN-04. *S. alfalfae* XN-04 shows a high similarity (≥ 75%) with 10 known clusters (Table 3), but none of these compounds have been reported for antifungal activity. Thus, the antifungal metabolites of XN-04 may be new or may have another biosynthesis pathway or uncharacterized mechanism. Further studies revealed that the EtOAc extract of XN-04 inhibited the growth of *Fov* at very low concentrations. The antifungal activity of EtOAc extract indicated no significant reduction after treatment with heat and proteinase K, suggesting that the antifungal metabolites of XN-04 may be not proteins. A total of 29 compounds from MeOH extract were identified by GC–MS in our present study (Table S10), among which (1) n-hexadecanoic acid, (2) 9,12-octadecadienoic acid (Z,Z)-, methyl ester, (3) 9,12-octadecadienoic acid (Z,Z)- (4) 9-octadecenoic acid, (Z)-, and (5) bis(2-ethylhexyl) phthalate have been reported with antifungal activity in previous studies (Walters et al., 2004; Rahman and Anwar, 2006; Pinto et al., 2017;Qi et al., 2019). Therefore, we hypothesize that XN-04 might control *Fov* using multiple metabolites. To date, it is still unclear whether these compounds are related to the biocontrol ability of XN-04, which merit further investigation in the future study.

### The Mechanisms of XN-04 Promoting Plant Growth

Many BCAs have the characteristics of promoting plant growth and facilitating the absorption and utilization of mineral nutrients, which confers them additional advantages in the disease suppression process (Olanrewaju and Babalola, 2019). In this study, XN-04 was demonstrated to effectively promote cotton growth under greenhouse conditions. Previous study has documented that the secretion of IAA and ACC deaminase is very important for the growth-promoting activity of *Streptomyces* spp. (Vurukonda et al., 2018). El-Tarabily (2008) reported that the increased growth promoted by *S. filipinensis*, when compared to *S. atrovirens*, was due to the production of both IAA and ACC, whereas *S. atrovirens* only produced ACC deaminase.

*Streptomyces alfalfae* XN-04 also exhibited other interesting PGP activities, such as siderophore production and phosphate solubilization. Siderophore compounds are potential plant growth promoters and disease suppressors. *Streptomyces* spp. was reported to produce hydroxymate-type siderophores, which inhibit the growth of phytopathogens by limiting iron in the rhizosphere (Khamna et al., 2009). Notably, KEGG together with antiSMASH analysis showed that XN-04 exhibited the great potential to synthesize multiple types of siderophore, i.e., enterobactin, coelichelin, mirubactin, and a NRPS-independent siderophore. The existence of siderophores with various chelating groups in the same strain can benefit the microorganism itself, thus improving its competitiveness in the environment. It can be presumed that the suppression of plant growth-promoting by XN-04 might be due to these mechanisms, but further studies are required to provide evidence regarding the actual mechanisms.

## Data Availability Statement

The datasets presented in this study can be found in online repositories. The names of the repository/repositories and accession number(s) can be found in the article/Supplementary Material.

## Author Contributions

JC and YW designed the study and wrote the manuscript. JC, LH, NC, and RJ performed the experiments. JC, LH, NC, and YW analyzed the data. QM helped to revise the manuscript. All authors contributed to the article and approved the submitted version.

## Funding

This work was supported by the National Key R&D Program of the Ministry of Science and Technology (2018YFD0201205-2) and the Key Industry Chain Innovation Project of Shaanxi province (2020ZDLNY07-02).

## Conflict of Interest

The authors declare that the research was conducted in the absence of any commercial or financial relationships that could be construed as a potential conflict of interest.

## Publisher’s Note

All claims expressed in this article are solely those of the authors and do not necessarily represent those of their affiliated organizations, or those of the publisher, the editors and the reviewers. Any product that may be evaluated in this article, or claim that may be made by its manufacturer, is not guaranteed or endorsed by the publisher.
