# Supplementary material for: The Biocontrol and Plant Growth-Promoting Properties of Streptomyces alfalfae XN-04 Revealed by Functional and Genomic Analysis
Source: Front Microbiol. 2021 Sep 22;12:745766. doi: 10.3389/fmicb.2021.745766 (PMC8493286; doi:10.3389/fmicb.2021.745766)
Supplement: Supplementary file 1 [file Data_Sheet_1.docx]

Supplementary Material

##
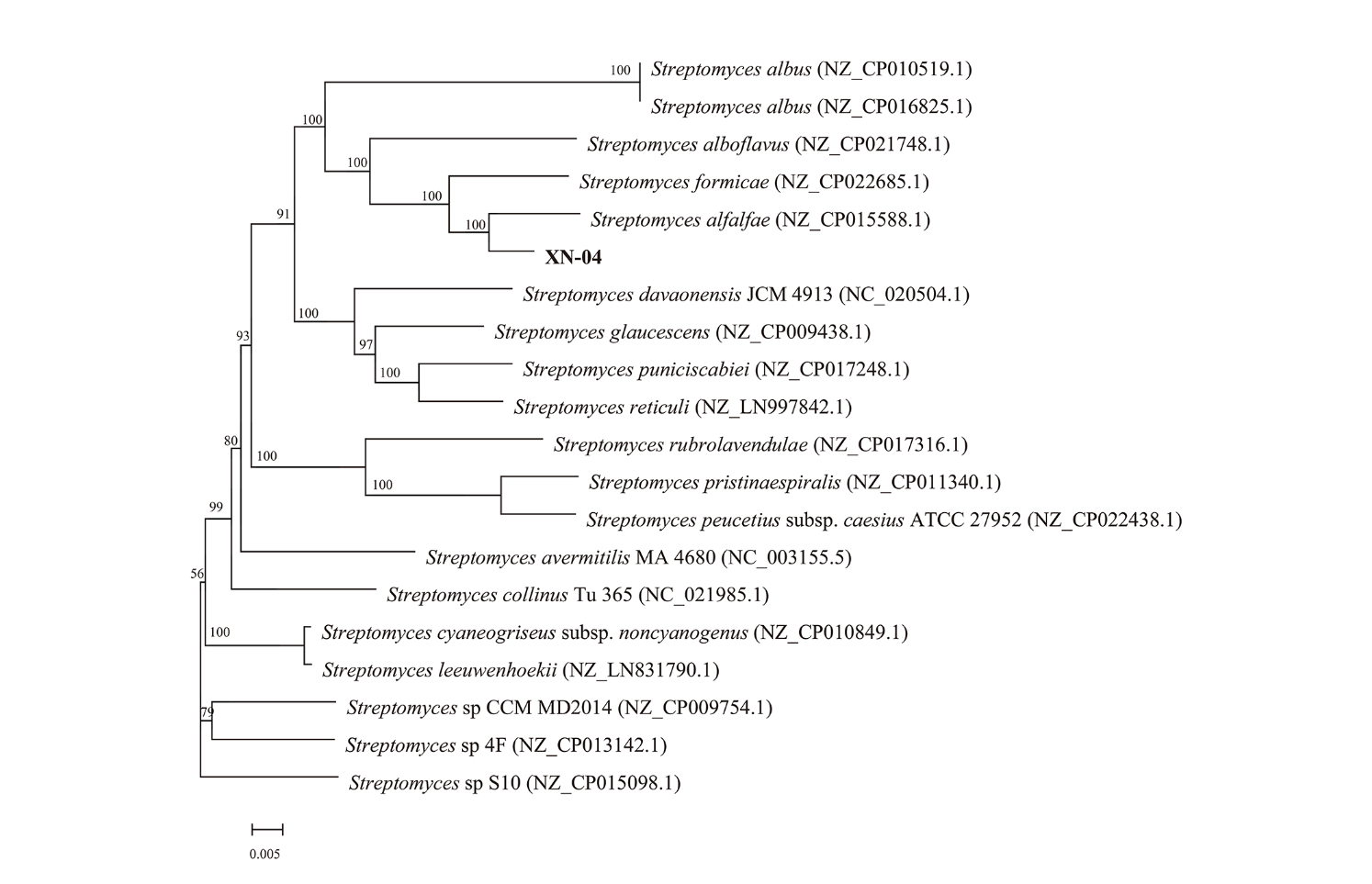
Supplementary Figures

**Supplementary Figure 1.** A phylogenetic tree of XN-04 with the other completely sequenced representatives of *Streptomyces* spp. strains using 31 housekeeping genes (*dnaG, frr, infC, nusA, pgk, pyrG, rplA, rplB, rplC, rplD, rplE, rplF, rplK, rplL, rplM, rplN, rplP, rplS, rplT, rpmA, rpoB, rpsB, rpsC, rpsE, rpsI, rpsJ, rpsK, rpsM, rpsS, smpB, tsf*) as phylogenetic markers. The significance of each branch is indicated by a bootstrap value calculated for 1000 subsets.

**
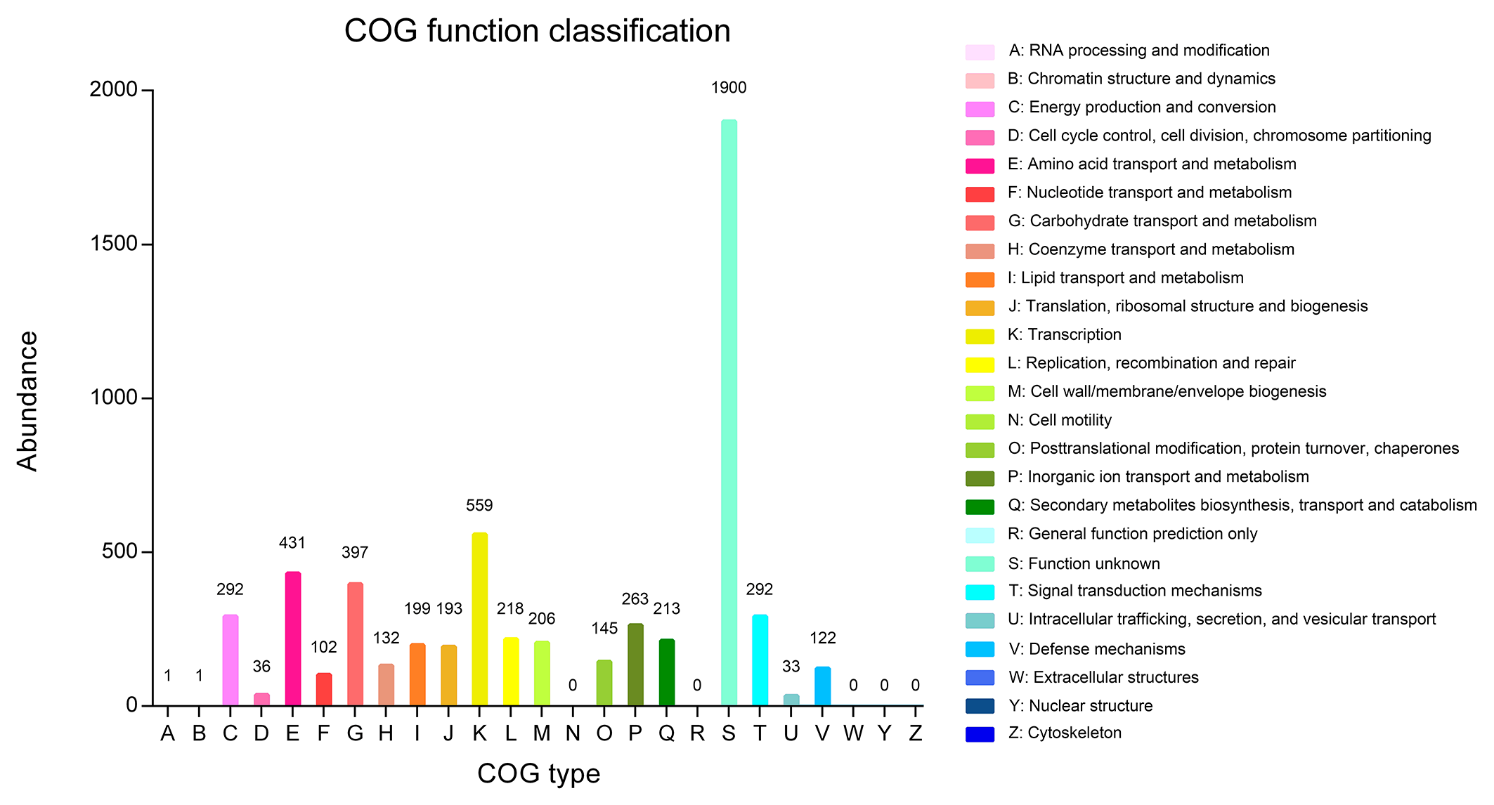
Supplementary Figure 2.** COG functional categories of *S. alfalfae* XN-04.

**
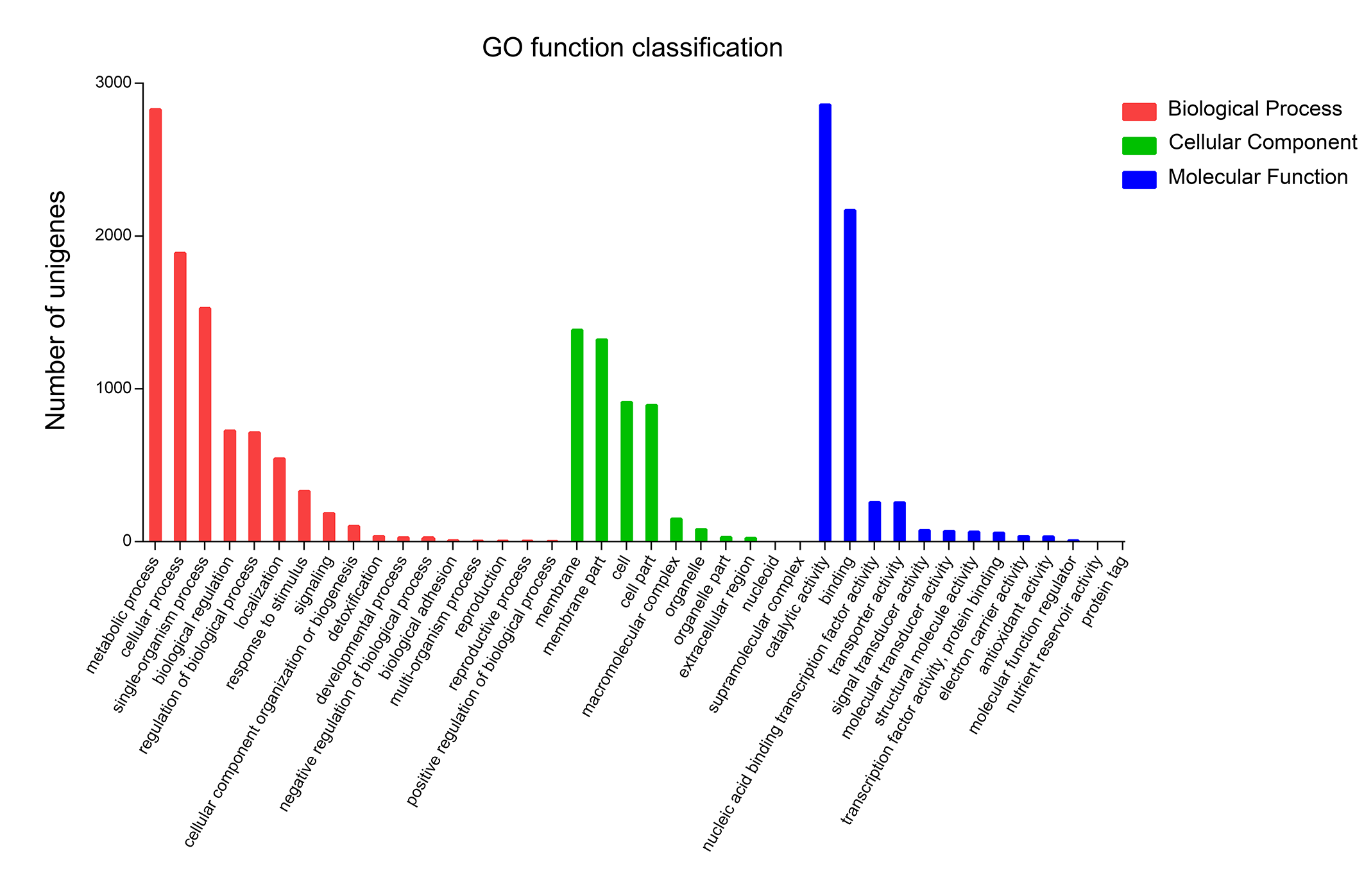
Supplementary Figure 3.** GO functional categories of *S. alfalfae* XN-04.


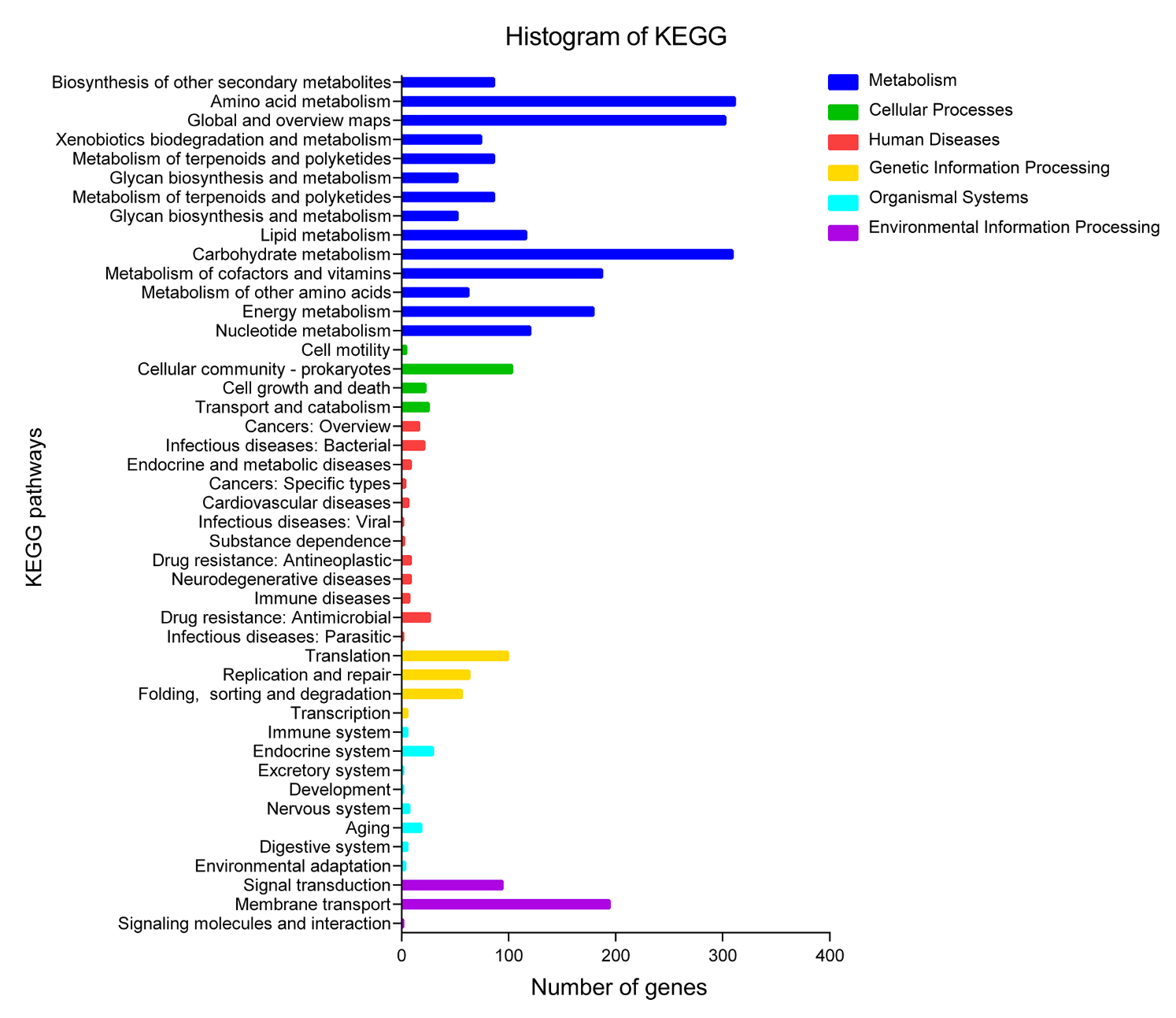
**Supplementary Figure 4.** KEGG functional categories of *S. alfalfae* XN-04.


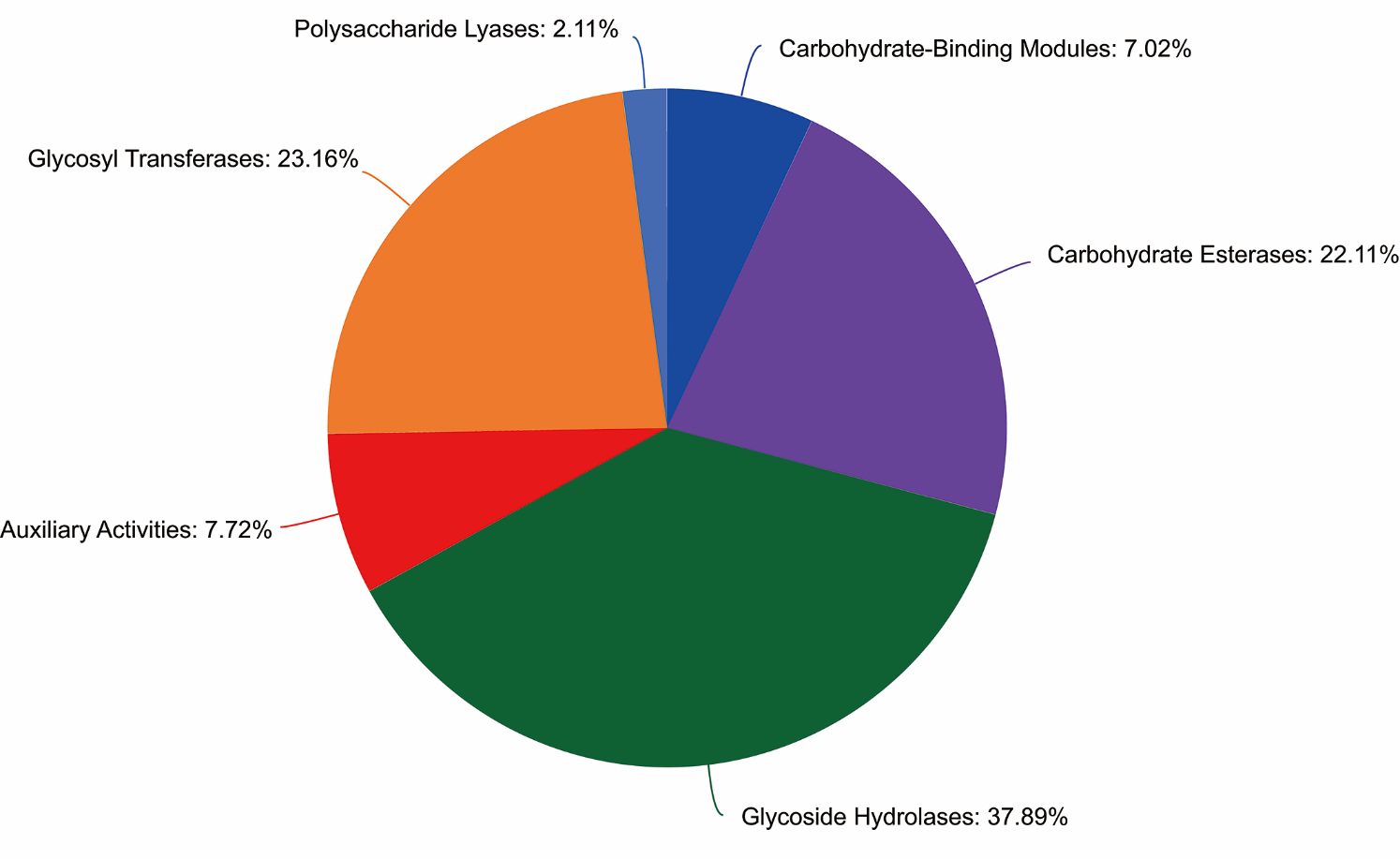
**Supplementary Figure 5.** Gene count distributions of carbohydrate-active enzyme (CAZy) families.


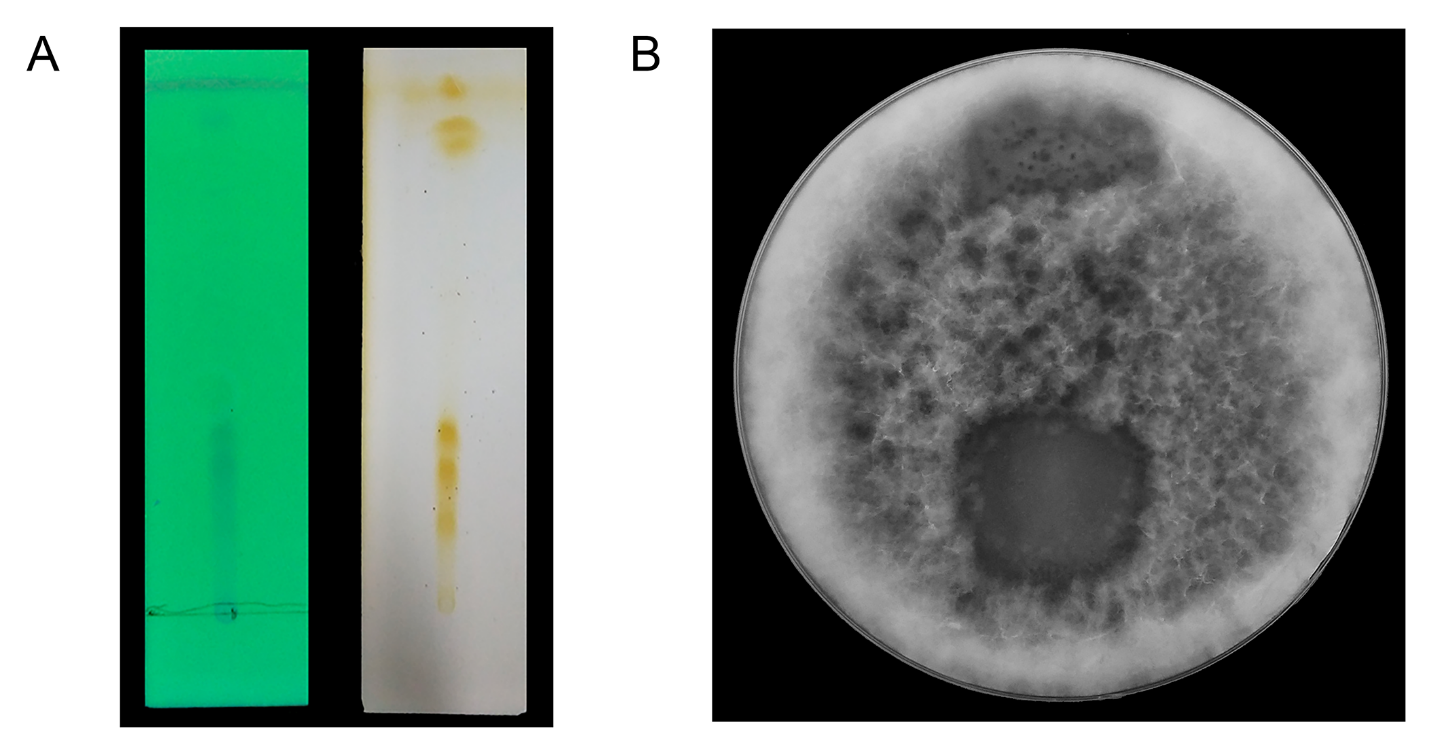
**Supplementary Figure 6.** Thin layer chromatography (TLC) of the EtOAc extract. (A) With UV_254_ (left) and iodine (right). (B) Bioautography of the EtOAc extract against *Fov*.

## Supplementary Tables

**Table S1.** Inhibition radius of the isolated actinomycetes against *Fov.*

| Isolate No. | Inhibition radius (mm) | Isolate No. | Inhibition radius (mm) |
| --- | --- | --- | --- |
| GS-01 | 10.75 | XN-07 | 5.50 |
| GS-02 | - | XN-08 | - |
| GS-03 | 13.25 | XN-09 | - |
| GS-04 | - | XN-10 | - |
| GS-05 | - | XN-11 | - |
| GS-06 | 6.00 | XN-12 | - |
| GS-07 | 1.75 | XN-13 | 8.00 |
| GS-08 | - | XN-14 | - |
| GS-09 | - | XN-15 | - |
| GS-10 | - | XN-16 | 5.00 |
| XN-01 | 10.00 | XN-17 | 5.00 |
| XN-02 | 3.00 | XN-18 | - |
| XN-03 | 4.50 | XN-19 | 4.50 |
| XN-04 | 13.50 | XN-20 | - |
| XN-05 | 5.50 | XN-21 | 3.25 |
| XN-06 | - | XN-22 | 2.00 |

Note: “-” indicates that the inhibition activity of the strain against *Fov* was not detected.

**Table S2.** Culture characteristics of *S. alfalfae* XN-04.

| Medium | Growth situation | Aerial mycelium color | Substrate mycelium color | Soluble pigment |
| --- | --- | --- | --- | --- |
| ISP 1 | + | Light yellow | Light yellow | - |
| ISP 2 | + | Yellow | Yellow | - |
| ISP 3 | +++ | Light pink | Yellow | - |
| ISP 4 | + | Yellow | Yellow | - |
| ISP 5 | + | White | Yellow | - |
| ISP 6 | ++ | Yellow | Yellow | - |
| ISP 7 | + | White | Yellow | - |
| MS | +++ | Light pink | Yellow | - |
| GS | + | Yellow | Yellow | - |
| PDA | +++ | Light pink | Yellow | - |

ISP 1: tryptone-yeast extract agar; ISP 2: yeast extract-malt extract agar; ISP 3: oatmeal agar; ISP 4: inorganic salts-starch agar; ISP 5: glycerol-asparagine agar; ISP 6: peptone-yeast extract agar; ISP 7: tyrosine agar.

Note: “+”: Growth; “++”: Moderate growth; “+++”: Abundant growth.

**Table S3.** Physiological and biochemical properties of *S. alfalfae* XN-04

| Test | Result | Test | Result |
| --- | --- | --- | --- |
| Carbon sources utilization: | | Nitrogen sources utilization: | |
| Glucose | + | NH_4_NO_3_ | + |
| Fructose | + | Ca(NO_3_)_2_ | + |
| Maltose | + | NH_4_Cl | + |
| Galactose | + | Urea | + |
| Sucrose | + | Glycine | + |
| Arabinose | + | KNO_3_ | + |
| Rhamnose | + | Glusate | + |
| Inositol | + |  |  |
| Mannitol | - |  |  |
| Biochemical test: | | Growth with / at: | |
| Starch hydrolysis | + | NaCl tolerance | 0% - 8% (w/v) |
| Cellulose hydrolysis | - | Growth temperature (°C) | 5 - 35 |
| Melanin production | - |  |  |
| Gelatin liquefaction | + |  |  |
| Milk coagulation | + |  |  |
| Milk peptonization | + |  |  |

Note：“+” Positive reaction; “-” Negative reaction.

**Table S4.** Genes involved in the degradation of chitin, glucan and cellulose.

| Classification | Gene ID | Gene name | Family | EC | Predicted function |
| --- | --- | --- | --- | --- | --- |
| Chitin-related | gene1418 | *-* | GH18 | 3.2.1.14 | chitinase |
|  | gene1542 | *-* | GH18 | 3.2.1.14 | chitinase |
|  | gene1559 | *-* | GH18 | 3.2.1.14 | chitinase |
|  | gene3037 | *-* | GH18 | 3.2.1.14 | chitinase |
|  | gene5528 | *-* | GH18 | 3.2.1.14 | chitinase |
|  | gene5866 | *-* | GH18 | 3.2.1.14 | exochitinase |
|  | gene6977 | *-* | GH19 | 3.2.1.14 | chitinase |
|  | gene7110 | *-* | GH18 | 3.2.1.14 | chitinase |
|  | gene0187 | *cpbD* | AA10 | - | chitin-binding protein |
|  | gene0189 | *cpbD* | AA10 | - | chitin-binding protein |
|  | gene0328 | *cpbD* | AA10 | - | chitin-binding protein |
|  | gene0743 | *cpbD* | AA10 | - | chitin-binding protein |
|  | gene1960 | *cpbD* | AA10 | - | chitin-binding protein |
|  | gene3079 | *cpbD* | AA10 | - | chitin-binding protein |
|  | gene6439 | *csn* | GH46 | 3.2.1.132 | chitosanase |
|  | gene2997 | *nagZ* | GH3 | 3.2.1.52 | β-N-acetyl hexosaminidase |
|  | gene3023 |  | GH20 | 3.2.1.52 | hexosaminidase |
|  | gene3184 |  | GH20 | 3.2.1.52 | hexosaminidase |
|  | gene5018 |  | GH20 | 3.2.1.52 | hexosaminidase |
|  | gene5405 | *nagZ* | GH3 | 3.2.1.52 | β-N-acetyl hexosaminidase |
|  | gene6748 | *nagZ* | GH3 | 3.2.1.52 | β-N-acetyl hexosaminidase |
| Glucan-related | gene0711 | *-* | GH16 | 3.2.1.39 | endo-1,3-β-glucanase |
|  | gene0792 | *-* | GH16 | 3.2.1.39 | endo-1,3-β-glucanase |
|  | gene1239 | *-* | GH16 | 3.2.1.39 | endo-1,3-β-glucanase |
|  | gene6841 | *-* | GH64 | 3.2.1.39 | endo-1,3-β-glucanase |
| Cellulose-related | gene2229 | *-* | GH6 | 3.2.1.4 | endo-1,4-β-glucanase |
|  | gene3086 | *-* | GH6 | 3.2.1.4 | endo-1,4-β-glucanase |
|  | gene5565 | *-* | GH6 | 3.2.1.4 | endo-1,4-β-glucanase |
|  | gene6623 | *-* | GH6 | 3.2.1.4 | endo-1,4-β-glucanase |
|  | gene0190 | *cbhA* | GH6 | 3.2.1.91 | cellulose 1,4-β-cellobiosidase |
|  | gene6624 | *cbhA* | GH6 | 3.2.1.91 | cellulose 1,4-β-cellobiosidase |
|  | gene0605 | *bglX* | GH3 | 3.2.1.21 | β-glucosidase |
|  | gene2795 | *bglB* | GH1 | 3.2.1.21 | β-glucosidase |
|  | gene3036 | *bglB* | GH1 | 3.2.1.21 | β-glucosidase |
|  | gene6631 | *bglX* | GH3 | 3.2.1.21 | β-glucosidase |
|  | gene6979 | *bglX* | GH3 | 3.2.1.21 | β-glucosidase |

**Table S5.** Genes involved in the degradation of protein and lipids.

| Classification | Gene ID | Gene name | Predicted function |
| --- | --- | --- | --- |
| Protein-related | gene0779 |  | serine protease |
|  | gene2517 |  | site-2 protease family protein |
|  | gene2794 |  | metalloprotease |
|  | gene2918 |  | serine protease |
|  | gene3156 | *ina* | protease |
|  | gene3503 |  | M6 family metalloprotease |
|  | gene3857 |  | protease |
|  | gene3912 |  | protease PrsW |
|  | gene4123 | *pepD* | protease |
|  | gene4235 |  | rhomboid family intramembrane serine protease |
|  | gene4715 |  | protease |
|  | gene4819 | *htpX* | zinc metalloprotease HtpX |
|  | gene5245 |  | serine protease |
|  | gene5304 | *pepD* | protease |
|  | gene5611 | *sprC* | serine protease |
|  | gene5876 |  | zinc metalloprotease |
|  | gene5902 |  | serine protease |
|  | gene5969 |  | zinc metalloprotease |
|  | gene6018 |  | serine protease |
|  | gene6222 |  | protease |
|  | gene6287 |  | serine protease |
|  | gene6383 |  | CAAX protease |
|  | gene6756 |  | Clp protease |
|  | gene6771 |  | serine protease |
|  | gene7335 |  | protease |
|  | gene7555 |  | serine protease |
| Lipids-related | gene0357 |  | lipase |
|  | gene0503 |  | lipase |
|  | gene0690 |  | lipase |
|  | gene0982 |  | lipase |
|  | gene0993 |  | lipase |
|  | gene1078 |  | GDSL family lipase |
|  | gene1940 |  | lipase |
|  | gene2143 |  | lysophospholipase |
|  | gene2264 |  | GDSL family lipase |
|  | gene3367 |  | GDSL family lipase |
|  | gene4371 |  | lipase |
|  | gene4903 |  | lipase |
|  | gene4956 |  | lipase |
|  | gene6925 |  | lysophospholipase |
|  | gene7128 |  | lipase |

**Table S6.** Genes involved in the modulation of plant hormones.

| Gene ID | Gene name | Product | Pathway |
| --- | --- | --- | --- |
| gene5416 |  | 1-aminocyclopropane-1-carboxylate deaminase | ACC catabolism |
| gene2296 | *trpA* | tryptophan synthase alpha chain | L-tryptophan production |
| gene2297 | *trpB* | tryptophan synthase beta chain |  |
| gene2298 | *trpC* | indole-3-glycerol phosphate synthase |  |
| gene2302 | *trpE* | anthranilate synthase component I |  |
| gene2412 | *trpD* | anthranilate phosphoribosyltransferase |  |
| gene7425 | *trpE* | anthranilate synthase component I |  |
| gene7426 | *trpG* | anthranilate synthase component II |  |
| gene7435 | *trpB* | tryptophan synthase beta chain |  |
| gene1651 |  | tryptophan 2-monooxygenase | IAA production; IAM pathway |
| gene6506 |  | tryptophan 2-monooxygenase |  |
| gene6507 |  | tryptophan 2-monooxygenase |  |
| gene6527 |  | tryptophan 2-monooxygenase |  |
| gene6528 |  | tryptophan 2-monooxygenase |  |
| gene6530 |  | tryptophan 2-monooxygenase |  |
| gene0633 | *amiE* | amidase |  |
| gene0736 | *amiE* | amidase |  |
| gene7102 | *amiE* | amidase |  |
| gene4354 |  | nitrilase | IAA production; IAN pathway |
| gene0356 | *aofH* | monoamine oxidase | IAA production; TAM pathway |
| gene0768 | *aofH* | monoamine oxidase |  |
| gene1131 |  | aldehyde dehydrogenase |  |
| gene1927 |  | aldehyde dehydrogenase |  |
| gene4584 |  | aldehyde dehydrogenase |  |
| gene5069 |  | aldehyde dehydrogenase |  |

**Table S7.** Genes involved in iron transport and siderophore production.

| Gene ID | Gene name | Product | Pathway |
| --- | --- | --- | --- |
| gene7018 |  | ferric iron reductase | Ferric iron reduction |
| gene0783 | *entF* | enterobactin synthetase component F |  |
| gene3505 | *entA* | 2,3-dihydro-2,3-dihydroxybenzoate dehydrogenase | Siderphore production |
| gene3506 | *entC* | isochorismate synthase |  |
| gene3507 | *entE* | 2,3-dihydroxybenzoate-AMP ligase |  |
| gene3508 | *entB* | bifunctional isochorismate lyase / aryl carrier protein |  |
| gene6005 | *fes* | enterochelin esterase and related enzymes |  |
| gene0473 |  | iron complex transport system substrate-binding protein | Iron complex  transport |
| gene0474 |  | iron complex transport system permease protein |  |
| gene0475 |  | iron complex transport system ATP-binding protein |  |
| gene0684 |  | iron complex transport system ATP-binding protein |  |
| gene0685 |  | iron complex transport system permease protein |  |
| gene0686 |  | iron complex transport system substrate-binding protein |  |
| gene0862 |  | iron complex transport system permease protein |  |
| gene0863 |  | iron complex transport system substrate-binding protein |  |
| gene0864 |  | iron complex transport system ATP-binding protein |  |
| gene1200 |  | iron complex transport system ATP-binding protein |  |
| gene1201 |  | iron complex transport system permease protein |  |
| gene1202 |  | iron complex transport system permease protein |  |
| gene1203 |  | iron complex transport system substrate-binding protein |  |
| gene2013 |  | iron complex transport system ATP-binding protein |  |
| gene2014 |  | iron complex transport system permease protein |  |
| gene2015 |  | iron complex transport system permease protein |  |
| gene2029 |  | iron complex transport system ATP-binding protein |  |
| gene2465 |  | iron complex transport system ATP-binding protein |  |
| gene2466 |  | iron complex transport system permease protein |  |
| gene2467 |  | iron complex transport system substrate-binding protein |  |
| gene3022 |  | iron complex transport system substrate-binding protein |  |
| gene5627 |  | iron complex transport system ATP-binding protein |  |
| gene5997 |  | iron complex transport system permease protein |  |
| gene5998 |  | iron complex transport system permease protein |  |
| gene5999 |  | iron complex transport system ATP-binding protein |  |
| gene6000 |  | iron complex transport system substrate-binding protein |  |
| gene6647 |  | iron complex transport system substrate-binding protein |  |
| gene6648 |  | iron complex transport system ATP-binding protein |  |
| gene6649 |  | iron complex transport system permease protein |  |
| gene7252 |  | iron complex transport system substrate-binding protein |  |
| gene7345 |  | iron complex transport system substrate-binding protein |  |
| gene7348 |  | iron complex transport system permease protein |  |
| gene7349 |  | iron complex transport system ATP-binding protein |  |

**Table S8.** Genes involved in phosphate solubilization and transport.

| Gene ID | Gene name | Product | Pathway |
| --- | --- | --- | --- |
| gene3384 | *ppx-gppA* | exopolyphosphatase | Degradation of inorganic  polyphosphates |
| gene4648 | *ppx-gppA* | exopolyphosphatase |  |
| gene4591 | *ppa* | inorganic pyrophosphatase |  |
| gene1096 | *phoD* | alkaline phosphatase | Organic phosphate  solubilization |
| gene2327 | *phoD* | alkaline phosphatase |  |
| gene2533 | *phoD* | alkaline phosphatase |  |
| gene2807 | *phoH* | phosphate starvation-inducible protein PhoH |  |
| gene6931 | *phoD* | alkaline phosphatase |  |
| gene3807 | *phoU* | phosphate transport system regulatory protein PhoU | Phosphate transport |
| gene3871 | *pstS* | phosphate transport system substrate-binding protein |  |
| gene3872 | *pstC* | phosphate transport system permease protein |  |
| gene3873 | *pstA* | phosphate transport system permease protein |  |
| gene3874 | *pstB* | phosphate transport system ATP-binding protein |  |
| gene7145 | *pstS* | phosphate transport system substrate-binding protein |  |
| gene3090 | *phnS* | 2-aminoethylphosphonate transport system substrate-binding protein | Phosphonate transport |
| gene3091 | *phnV* | 2-aminoethylphosphonate transport system permease protein |  |
| gene3092 | *phnU* | 2-aminoethylphosphonate transport system permease protein |  |
| gene3093 | *phnT* | 2-aminoethylphosphonate transport system ATP-binding protein |  |

**Table S9.** Compounds identified from the MeOH extract of XN-04 through GC-MS.

| No. | Predicted compounds | RT (min) | MF | MM | CAS# | Activity | References |
| --- | --- | --- | --- | --- | --- | --- | --- |
| 1 | 1-Undecene | 8.67 | C_11_H_22_ | 154 | 821-95-4 |  |  |
| 2 | 2,5-Dimethylphenol | 9.93 | C_8_H_10_O | 122 | 95-87-4 |  |  |
| 3 | 5-Octadecyne | 13.07 | C_18_H_34_ | 250 | 71899-42-8 |  |  |
| 4 | 4a,5-Dimethylhexahydro-4H-1,3-benzodioxin-4-one | 15.92 | C_10_H_16_O_3_ | 184 | 124899-17-8 |  |  |
| 5 | Phenol, 2,4-bis(1,1-dimethylethyl) | 18.05 | C_14_H_22_O | 206 | 96-76-4 |  |  |
| 6 | N-Octadecylacrylamide | 20.93 | C_21_H_41_NO | 323 |  |  |  |
| 7 | Tetradecanoic acid, methyl ester | 21.61 | C_15_H_30_O_2_ | 242 | 124-10-7 |  |  |
| 8 | 1-(Dimethylamino)pyrrole | 21.96 | C_6_H_10_N_2_ | 110 | 78307-76-3 |  |  |
| 9 | Carbonic acid, monoamide, N-octadecyl-, propargyl ester | 22.31 | C_22_H_41_NO_2_ | 351 |  |  |  |
| 10 | Pentadecanoic acid, methyl ester | 23.46 | C_16_H_32_O_2_ | 256 | 7132-64-1 |  |  |
| 11 | (S)-12-Methyltetradecanoic acid methyl ester | 23.59 | C_16_H_32_O_2_ | 256 | 62691-05-8 |  |  |
| 12 | Pentadecanoic acid | 24.22 | C_15_H_30_O_2_ | 242 | 1002-84-2 |  |  |
| 13 | Hexadecanoic acid, methyl ester | 25.22 | C_17_H_34_O_2_ | 270 | 112-39-0 |  |  |
| 14 | Dibutyl phthalate | 26.32 | C_16_H_22_O_4_ | 278 | 84-74-2 |  |  |
| 15 | n-Hexadecanoic acid | 26.46 | C_16_H_32_O_2_ | 256 | 57-10-3 | Antifungal | Qi et al., 2019 |
| 16 | Hexadecanoic acid, 14-methyl-, methyl ester | 27.03 | C_18_H_36_O_2_ | 284 | 2490-49-5 |  |  |
| 17 | cis-10-Heptadecenoic acid, methyl ester | 27.25 | C_18_H_34_O_2_ | 282 |  |  |  |
| 18 | 9,12-Octadecadienoic acid (Z,Z)-, methyl ester | 28.56 | C_19_H_34_O_2_ | 294 | 112-63-0 | Antifungal | Pinto et al., 2017 |
| 19 | 9-Octadecenoic acid, methyl ester, (E)- | 28.67 | C_19_H_36_O_2_ | 296 | 1937-62-8 |  |  |
| 20 | Octadecanoic acid, methyl ester | 29.08 | C_19_H_38_O_2_ | 298 | 112-61-8 |  |  |
| 21 | 9,12-Octadecadienoic acid (Z,Z)- | 29.18 | C_18_H_32_O_2_ | 280 | 60-33-3 | Antifungal | Walters et al., 2004 |
| 22 | 9-Octadecenoic acid (Z)- | 29.28 | C_18_H_34_O_2_ | 282 | 112-80-1 | Antifungal | Walters et al., 2004 |
| 23 | Bis(2-ethylhexyl) adipate | 32.90 | C_22_H_42_O_4_ | 370 | 103-23-1 |  |  |
| 24 | Bis(2-ethylhexyl) phthalate | 34.64 | C_24_H_38_O_4_ | 390 | 117-81-7 | Antifungal | Rahman and Anwar, 2006 |
| 25 | 8,12-Tetradecadienoic acid, 5-ethenyl-3,5,9,13-tetramethyl-, methyl ester | 36.72 | C_21_H_36_O_2_ | 320 | 36237-73-7 |  |  |
| 26 | 2,3-Oxidosqualene | 37.05 | C_30_H_50_O | 426 | 7200-26-2 |  |  |
| 27 | Squalene | 37.36 | C_30_H_50_ | 410 | 111-02-4 |  |  |
| 28 | Clionasterol | 41.43 | C_29_H_50_O | 414 | 83-47-6 |  |  |
| 29 | Diploptene | 41.55 | C_30_H_50_ | 410 | 1615-91-4 |  |  |

**Table S10.** Colonization dynamics in the cotton rhizosphere by EGFP-labeled XN-04.

| Time (dai^1^) | Cell density of EGFP-labeled XN-04 (cfu/g root weight) |
| --- | --- |
| 2 | 3.43 × 10^4^ c |
| 4 | 3.38 × 10^5^ b |
| 7 | 1.33 × 10^6^ a |

^1^ dai: days after inoculation.
